# Variational autoencoding of gene landscapes during mouse CNS development uncovers layered roles of Polycomb Repressor Complex 2

**DOI:** 10.1093/nar/gkac006

**Published:** 2022-01-20

**Authors:** Ariane Mora, Jonathan Rakar, Ignacio Monedero Cobeta, Behzad Yaghmaeian Salmani, Annika Starkenberg, Stefan Thor, Mikael Bodén

**Affiliations:** School of Chemistry and Molecular Biosciences, University of Queensland, St Lucia, QLD 4072, Australia; Department of Clinical and Experimental Medicine, Linköping University, SE-58185 Linköping, Sweden; Department of Clinical and Experimental Medicine, Linköping University, SE-58185 Linköping, Sweden; Department of Physiology, Universidad Autonoma de Madrid, Madrid, Spain; Department of Clinical and Experimental Medicine, Linköping University, SE-58185 Linköping, Sweden; Department of Cell and Molecular Biology, Karolinska Institute, SE-171 65 Stockholm, Sweden; Department of Clinical and Experimental Medicine, Linköping University, SE-58185 Linköping, Sweden; Department of Clinical and Experimental Medicine, Linköping University, SE-58185 Linköping, Sweden; School of Biomedical Sciences, University of Queensland, St Lucia, QLD 4072, Australia; School of Chemistry and Molecular Biosciences, University of Queensland, St Lucia, QLD 4072, Australia

## Abstract

A prominent aspect of most, if not all, central nervous systems (CNSs) is that anterior regions (brain) are larger than posterior ones (spinal cord). Studies in *Drosophila* and mouse have revealed that Polycomb Repressor Complex 2 (PRC2), a protein complex responsible for applying key repressive histone modifications, acts by several mechanisms to promote anterior CNS expansion. However, it is unclear what the full spectrum of PRC2 action is during embryonic CNS development and how PRC2 intersects with the epigenetic landscape. We removed PRC2 function from the developing mouse CNS, by mutating the key gene *Eed*, and generated spatio-temporal transcriptomic data. To decode the role of PRC2, we developed a method that incorporates standard statistical analyses with probabilistic deep learning to integrate the transcriptomic response to PRC2 inactivation with epigenetic data. This multi-variate analysis corroborates the central involvement of PRC2 in anterior CNS expansion, and also identifies several unanticipated cohorts of genes, such as proliferation and immune response genes. Furthermore, the analysis reveals specific profiles of regulation via PRC2 upon these gene cohorts. These findings uncover a differential logic for the role of PRC2 upon functionally distinct gene cohorts that drive CNS anterior expansion. To support the analysis of emerging multi-modal datasets, we provide a novel bioinformatics package that integrates transcriptomic and epigenetic datasets to identify regulatory underpinnings of heterogeneous biological processes.

## INTRODUCTION

The embryonic central nervous system (CNS) is patterned along the anterior–posterior (A–P) axis, evident by e.g. the expression of brain-specific transcription factors (TFs) in anterior regions and the Hox homeotic genes in posterior regions. A–P patterning of the CNS has two key consequences: first, the generation of distinct cell types in different regions, and second, the striking expansion of the brain relative to the spinal cord. Studies in *Drosophila* have revealed that anterior CNS expansion is driven by a longer phase of neural progenitor proliferation, more prevalent daughter cell divisions and faster cell cycle speeds in anterior regions, combining to generate much larger average lineages anteriorly (1). This A-P ‘stemness’ gradient further manifests, and is driven, by an A–P gradient of neural progenitor TF (e.g. SoxB family) and cell cycle gene expression (1–3). These expression gradients are in turn promoted by the selective expression of the A–P patterning TFs (3–5). However, it is unclear if the principles uncovered in *Drosophila* are fully conserved in mammals.

The selective expression of TFs along the A-P axis is under control of epigenetic cues, where the Polycomb Repressive Complex 2 (PRC2) plays a prominent role (6). PRC2 mono-, di- and tri-methylates Histone 3 upon residue Lysine 27 (H3K27me1/2/3), typically resulting in proximal gene repression (7,8). Inactivating PRC2 during *Drosophila* or vertebrate CNS development, by mutating either one of the core complex genes *Ezh2* or *Eed* (*DrosophilaE(z)* and *esc*, respectively), induces ectopic expression of Hox genes in the anterior CNS (5,9), and reduces brain-specific TFs expression (4,5). PRC2 inactivation leads to undergrowth of the anterior CNS (5,9–12), while not affecting the spinal cord growth (5). The reduced brain growth following PRC2 inactivation appears to be, at least in part, due to reduced proliferation, in particular of daughter cells (5,13). The reduced proliferation observed in both mouse and *Drosophila* PRC2 mutants appears to result from (I) the down-regulation of brain-specific TFs, (II) upregulation of Hox genes, and (III) downregulation of neural progenitor stemness genes, (IV) decreased expression of pro-proliferative genes and (V) increased expression of anti-proliferative genes (2,4,5). However, it is unclear if PRC2 acts directly and/or indirectly upon the five gene groups associated with these roles, what the full spectrum of PRC2 action is during embryonic CNS development, and how PRC2 intersects with the epigenetic landscape.

Data integration across multi-modal datasets typically occurs after statistical tests have been used to group data points, referred to as ‘late integration’. However, this approach can obscure inter-dataset dependencies. In contrast, ‘early integration’ aims to retain these dependencies, by identifying salient patterns across datasets prior to statistical analysis (14,15). However, a lack of generalisability, interpretability and capacity to manage realistic scales of data have so far hindered widespread use of early integration across modalities (16).

Motivated by the need to integrate multiple epigenetic marks to label the chromatin landscape, ChromHMM (Chromatin Hidden Markov Model) uses a multi-variate hidden Markov model (17). The model is trained from genome-wide assays, such as ChIP-seq of histone modifications, across conditions to capture latent chromatin states manifested in co-occurring marks. ChromHMM was recently used to recover two distinct states that implicate H3K27me3 during mouse embryonic development (18). However, as the transcriptome was not incorporated at an early stage it is unclear how ChromHMM chromatin states relate to gene expression during development. Moreover, how distinct chromatin states support the biological heterogeneity and A–P patterning of the CNS was not investigated.

Variational autoencoders (VAEs) (19) are generative latent variable models able to encode relationships in mixed and multi-modal data types (20–22). VAEs learn to map data into a lower-dimensional space akin to methods such as principal component analysis (PCA), uniform manifold approximation and projection (UMAP) (23), t-distributed stochastic neighbour embedding (tSNE) (24) and potential of heat-diffusion for affinity-based transition embedding (PHATE) (25). Unlike these methods, which use eigen decomposition or neighbour embeddings to perform dimensionality reduction, VAEs cross features via successive layers of representation with ‘deep’ learning, a process that captures dependencies *between* data types, which in turn are accessed via latent variables. VAEs have been applied to interpret single cell RNA-seq (26–29), bulk RNA-seq (30,31), DNA methylation arrays (32) and histone modification ChIP-seq (33).

VAEs have also been used to integrate multi-omic data where each data point represents the genome-wide, multi-modal state of a patient (31). Indeed, the ability of VAEs to learn meaningful embeddings from high-dimensional data has sparked the development of a number of tools to analyse single cell data (29,34,35); all of these approaches map selected genome-wide observations to a heavily reduced latent space. Similarities at different levels, i.e. between individual and between successively larger groups of data points, have been shown to be preserved in the latent space to a degree not seen in comparable approaches (36). Here, we take a novel angle but still leveraging such capabilities: to identify genes co-*regulated* in time and space, and uncover specific regulatory cues in resulting states, we turn the data matrix ‘on its side’. A gene is represented as a data point defined by a set of experimental features collected at different times and in different tissues. Hence, the VAE is tasked to extract patterns evident in groups (or ‘cohorts’) of genes that are representative of specific biological function. Here, we sought to identify what regulatory cues PRC2 deploys, and ultimately explain the undergrowth of brains when PRC2 is inhibited.

To understand the role of PRC2 in establishing the CNS A–P axis we generated 64 transcriptomes from wild type (WT) and PRC2 knock-out (*Eed-cKO*) mouse embryos, at different developmental stages, and from the forebrain (FB), midbrain (MB), hindbrain (HB), and spinal cord (SC) regions of the CNS. We developed a workflow to analyse these data, which involves three stages: (i) differential analysis of transcriptomes; (ii) statistical analysis of genes stratified by expression changes and wild type histone modification data; (iii) VAE analysis to extract latent gene descriptors from transcriptomic and epigenetic data. The VAE analysis identified five functionally distinct gene cohorts with shared dependency on PRC2: (i) posterior genes, (ii) anterior genes, (iii) development genes, (iv) proliferation genes and (v) immune response genes. Surprisingly, analysis of the mode of regulation for each gene cohort reveals that while the first three cohorts appear primarily directly regulated, the latter two display a mix of direct and in-direct regulation by PRC2. Thus, our novel integrative approach identifies and stratifies genes by mode of regulation across CNS development.

## MATERIALS AND METHODS

### In vivo mouse models

*Eed* ^*fl*/*fl*^ (37) was obtained from the Jackson Laboratory Stock Center (Bar Harbor, Maine; stock number #022727). *Sox1-Cre* (38) was provided by J. Dias and J. Ericson, Karolinska Institute, Stockholm. Both lines were maintained on a B6:129 background. Mice were housed at the Linköping University animal facility in accordance with regional animal ethics regulations (Dnr 69-14). Pregnant females were sacrificed and embryos dissected between stages E11.5 and E18.5. Primers used for genotyping were: Cre1: GCG GTC TGG CAG TAA AAA CTA TC. Cre2: GTG AAA CAG CAT TGC TGT CAC TT. Eed1: GGG ACG TGC TGA CAT TTT CT. Eed2: CTT GGG TGG TTT GGC TAA GA. Sixteen mouse embryos were extracted from 8 female mice (16 *Eed*^*fl*/*fl*^). Two embryos were extracted from each mouse at E11.5, E13.5, E15.5, and E18.5 respectively.

### RNA-seq

Mouse embryos (E11.5, E13.5, E15.5 and E18.5) were dissected to extricate the CNS (the posterior-most part of the SC was not included). The CNS was then cut into four pieces, FB, MB, HB and SC. The E18.5 embryos were killed by decapitation, and then dissected (in line with ethical permits and regulations). The samples were stored at −80°C until RNA isolation, using Qiagen RNeasy Mini kit Cat.74104. RNA sequencing library preparation used the NEBNext Ultra RNA Library Prep Kit for Illumina by following manufacturer’s recommendations (NEB, Ipswich, MA, USA). The sequencing libraries were multiplexed and clustered. Samples were sequenced on Illumina HiSeq 2500, using a 50 bp Single End (SE) read configuration for E13.5 embryos, 150 bp Paired End (PE) read configuration for E11.5, E15.5 and E18.5, with a depth of ∼50–60 million reads (GeneWiz, New Jersey, NJ, USA). The RNA-seq files are available at GEO (GSE123331). Samples from the same age were litter mates, to ensure that the WT and *Eed-cKO* are as close as possible stage-wise.

### Immunohistochemistry

Embryos were fixed for 18–36 h in fresh 4% PFA at 4°C. After this they were transferred to 30% sucrose at 4°C until saturated. Embryos were embedded and frozen in OCT Tissue Tek (Sakura Finetek, Alphen aan den Rijn, Netherlands) and stored at −80°C. 20 and 40 μm cryosections were captured on slides, and treated with 4% fresh PFA for 15 min at room temperature. They were thereafter blocked and processed with primary antibodies in PBS with 0.2% Triton–X100 and 4% horse serum overnight at 4°C. Secondary antibodies, conjugated with AMCA, FITC, Rhodamine-RedX or Cy5, were used at 1:200 (Jackson ImmunoResearch, PA, USA). Slides were mounted in Vectashield (Vector, Burlingame, CA, USA). Primary antibodies were: Goat α-Sox2 (1:250, #SC-17320, Santa Cruz Biotechnology, Santa Cruz, CA, USA), Rabbit α-H3K27me3 (1:500, #9733, Cell Signaling Technology, Leiden, Netherlands), Isolectin GS-IB4-ALEXA647 conjugate (‘IB4’) (5–20 μg/ml, #I32450, Molecular Probes, Thermo Fisher Scientific, Waltham, MA, USA), Rabbit anti-Pax2 (1:100, #ab232460, Abcam, Cambridge, UK). IB4 and DAPI were included in the secondary antibody solutions. Confocal microscopes (Zeiss LSM700 or Zeiss LSM800) were used for fluorescent images. Confocal series were merged using LSM software or Fiji software (39). Images and graphs were compiled in Adobe Illustrator. The ratios between Sox2, IB4 and DAPI staining were calculated by taking the 100 × 500 × z-stack μm^3^ of staining volume for each marker in each stack of images, along the telencephalic ventricle and thresholded via ImageJ manual methods; ‘Huang’ for DAPI and ‘Moments’ for Sox2 and IB4.

### RNA-seq processing

FastQC (https://www.bioinformatics.babraham.ac.uk/projects/fastqc/) (version 0.11.9) was used to perform quality control (QC), along with multiQC (40) (version 1.8). The PE samples contained adapter content thus were trimmed using cutadapt (41) (version 2.10). Adapters used for trimming were: AGATCGGAAGAGCACACGTCTGAACTCCAGTCA (read 1) and AGATCGGAAGAGCGTCGTGTAGGGAAAGAGTGT (read 2), these were trimmed with an error tolerance of 5%, overlap of 3, and minimum Phred quality of 20. FastQC on the trimmed sequences passed QC for adapter content. RNA-seq data were then aligned to the mm10 genome using Hisat2 (42) (version 2.1.0), mm10 index was generated using the Hisat2 scripts. Reads from E13.5 were aligned using default parameters for SE reads (-U), with the other time points using default parameters for PE reads, the only parameters changed were: number of seeds set to 5; and number of primary alignments (*k*) also set to 5. Hisat2 reported an overall alignment rate >90% for all files. Reads were sorted using samtools (43) (version 1.10). FeatureCounts from subread (11) was used to count the reads mapping to genes. Exon feature was used for both SE and PE reads. The PE reads were aligned such that pair fragments with both ends successfully aligned were counted without considering the fragment length constraint and excluding chimeric fragments (-p -C -B -t exon -T). Default parameters were used for the E13.5 reads (-t exon). FeatureCounts reported an average mapping to genes of ∼70% for PE and ∼60% for SE.

### Differential expression

Differential expression analysis was performed using DESeq2 (44) (version 1.28.1), R (version 4.0.2). Genes were filtered if they had less than 10 counts in half of the samples. DE analysis was performed between each tissue, in each condition, using tissue as a factor and time as a batch factor. Then, for each tissue we used the three later time points, E13.5, E15.5 and E18.5, for differential expression between *Eed-cKO* and WT. E11.5 samples were omitted from DE as H3K27me3 is gradually lost between E10.5-E11.5 (5). We performed a similar analysis on the time points, grouping anterior tissues (FB, MB) and posterior tissues (HB, SC), resulting in eight DE analyses on WT versus *Eed-cKO* for time points using the tissue as a batch factor. Results were considered significant if a gene had an adjusted *P*-value (Benjamini–Hochberg (FDR-BH)) of less than or equal to 0.05. Py-venn (https://pypi.org/project/venn/) (version 0.1.3) and matplotlib-venn (https://pypi.org/project/matplotlib-venn/) (version 0.11.5) were used for displaying Venn diagrams and seaborn (45) (version 0.10.0) was used for all other visualisations. *Sox1-3*, FB, MB, HB and SC markers (Supplementary Table S1) were annotated on volcano plots if they were significant (*P* < 0.05) and exhibited an absolute log_2_FC >1.5.

### ChIP-seq processing

ChIP-seq data (NarrowPeak files, IDR reproducible peaks selected) for mm10 mouse FB, MB and HB tissues at embryonic time points were downloaded (November 2019) from ENCODE (46). Peaks were annotated to entrez (47) (NCBI, database) gene IDs by using scie2g (version 1.0.0), and scibiomart (version 1.0.0) (both developed as part of the package we publish with this paper) using the annotation (mmusculus Ensembl GRCm38) from Biomart, Ensembl (48). Peaks were assigned to a gene if they were located within 2.5 kB upstream of the TSS or within 500 bp of the gene body, except for H3K36me3 which was assigned if it fell on the gene body (upstream 2.5 kB of the TSS and 500 bp window after the gene ends). Peaks were retained if their adjusted *P*-value was <0.05. If multiple peaks were assigned to a gene then the peak with the greatest signal was retained. Signal and widths were recorded for each peak. If no peak was mapped to a gene, this gene was assigned a zero value. Annotations from Gorkin *et al.* (18). were assigned to genes when overlapping the TSS (±10 bp). If a gene had multiple annotations, the first one was considered, thereby reducing the number of annotated genes (by Ensembl ID) from 53 254 to 52 772. These were then mapped to Entrez IDs. Fisher’s Exact test in scipy (49) (version 1.5.3) was used to compare annotations between a foreground and background dataset, *P*-values were adjusted for multiple tests using statsmodels (50) (0.12.1) package, with alpha as 0.1 and FDR-BH correction used.

### Label stratified analysis

Integration was performed in Python (version 3.8.2). Code and visualisations are made available and documented as a fully executable Jupyter Notebook (Jupyter Core 4.6.3). Analysis results are fully reproduced by stepping through the Notebook. Pandas (51) (version 1.0.3) was used to merge the FeatureCounts files on Entrez gene ID, yielding a dataset of 27 179 rows. Gene names were annotated to merged data frame using Ensembl mappings from Entrez to gene name, from this, there were 6279 genes without gene names (predicted or nc-RNA), which were omitted from the subsequent analysis, leaving 20 900 genes. RNA-seq data were normalised by using EdgeR’s (52) (version 3.30.3) TMM method, the log_2_ + 1 was then taken of the TMM counts using numpy (53) (version 1.18.2). Peak data were merged on assigned Entrez ID as per the ChIP-processing section above.

We performed a simple stratification to annotate genes based on changes in expression and repressive mark presence. We labelled each gene as unaffected, partly affected or consistently affected, by using the expression response to PRC2 in-activation as per the DE analyses. Partly affected genes refers to expressed genes (average TMM > 0.5 in either WT or *Eed-cKO*) displaying a significant difference in expression between WT and *Eed-cKO* in at least one of the DE analyses of FB, MB, HB, SC, or the anterior temporal analyses (E11.5, E13.5, E15.5, E18.5). Consistently affected refers to genes meeting the partly affected requirement *and* exhibiting an absolute log_2_FC >1.0 in ≥3 of the eight DE analyses. We then labelled each gene as marked or unmarked based on the presence of a H3K27me3 ChIP-seq peak in WT FB, MB, or HB E16.5 within 2.5 kB of the TSS.

### VAE analysis

VAEs are implemented in scivae (developed by us for this project) (version 1.0.0), which in turn uses Tensorflow (version 2.3.1) (54) and Keras (https://keras.io/) (version 2.4.3). VAEs were created using the consistently affected genes as input (randomly sub-divided into a training set with 85% genes). Input was the normalised transcriptome (64 features), the log_2_ of the H3K27me3 signal (21 features) and the log_2_FC from the DE analyses was used (12 features). All input data were scaled between 0 and 1. Mean squared error was used as the loss metric, with Maximum Mean Discrepancy (MMD) kernel as the distance between distributions for the sampling function, with a MMD weight of 1.0. The VAE was trained for 250 epochs using a batch size of 50. Different numbers of latent nodes were tested, ranging from 1 to 32. Selu activation functions were used for the first input and final output layers with Relu used for internal layers; adam optimiser was used with parameters: beta1 = 0.9, beta2 = 0.999, decay = 0.01, and a learning rate of 0.01. Gene cohorts were calculated for each latent dimension from the 3 node, consistently affected dataset, with genes having a value ±1.25 standard deviation (SD) from the mean (0). This resulted in six gene cohorts (two for each node) with 62, 180, 80, 223, 165 and 121 genes, respectively, these were used in subsequent functional analyses.

We compared VAE to PCA and tSNE from scikit-learn (version 1.0.1), UMAP from umap-learn (23) (version 0.4.2), PHATE from phate (25) (version 1.0.7). Default parameters were used, except for changing the number of components (3 or 6) and for tSNE, running ‘method=exact’, when n_components = 6. Given tSNE, UMAP, PHATE and the VAEs may vary in terms of projection based on a seed, 20 runs were evaluated. To evaluate how well methods grouped genes, we downloaded gene sets from AmiGO (55) associated with ‘Positive regulation of proliferation’, ‘Spinalcord development’, ‘Hindbrain development’, ‘Midbrain development’, and ‘Forebrain development’. The resulting gene lists were filtered to contain genes that were uniquely annotated with one term. We use the Silhouette score from sklearn to compute how well genes with each term were separated from genes with any other term, for each tool, in *D* = 3 and *D* = 6. The same test was performed with both consistently affected and all affected genes.

### Functional and statistical analysis

Over representation analysis on the gene cohorts was performed in R using enrichGO from clusterprofiler (56), (version 3.16.1). Entrez IDs were used and BH correction with FDR alpha of 0.1, using all GO annotations. Gene set enrichment analysis was performed using fgsea (57) (version 1.14.0).

Statistical tests use Mann–Whitney *U* with Bonferroni correction (from statsmodels, (50)) unless stated otherwise and use the following *P*-value annotation: NS: 0.05 < *P* ≤ 1, *: 0.01 < *P* ≤ 0.05, **: 0.001 < *P* ≤ 0.01, ***: 0.0001 < *P* ≤ 0.001****: *P* ≤ 0.0001. Human gene expression data (RPKM) were downloaded from PsychEncode (March 2021) (58), and mapped to mouse genes using biomart. Genes were matched if there was $>80\%$ similarity between the mouse homolog and the human gene. Only embryonic samples were considered and these were grouped into the following age groups W1–W4 (W1: 8–9 postconceptional weeks (PCW), W2: 12–13 PCW, W3: 14–18 PCW, W4: 18–22 PCW) (58).

### Reproducible, generative methods applicable for other dynamic systems

Our model of the developing mouse CNS is available as a downloadable package where the profile of any mouse gene can be queried in terms of its PRC2 response, in addition to an interactive website (http://bioinf.scmb.uq.edu.au:81/cnsvae/static/). We also provide a Python package with tutorials in R and Python for using the VAE for other dynamic systems where researchers are interested in integrating epigenetic and expression information. Our packages have been optimised for reproducibility by enabling saving of the VAE state, visualisation, and logging.

## RESULTS

### PRC2 is critical for the anterior–posterior CNS axis

**Figure 1.**
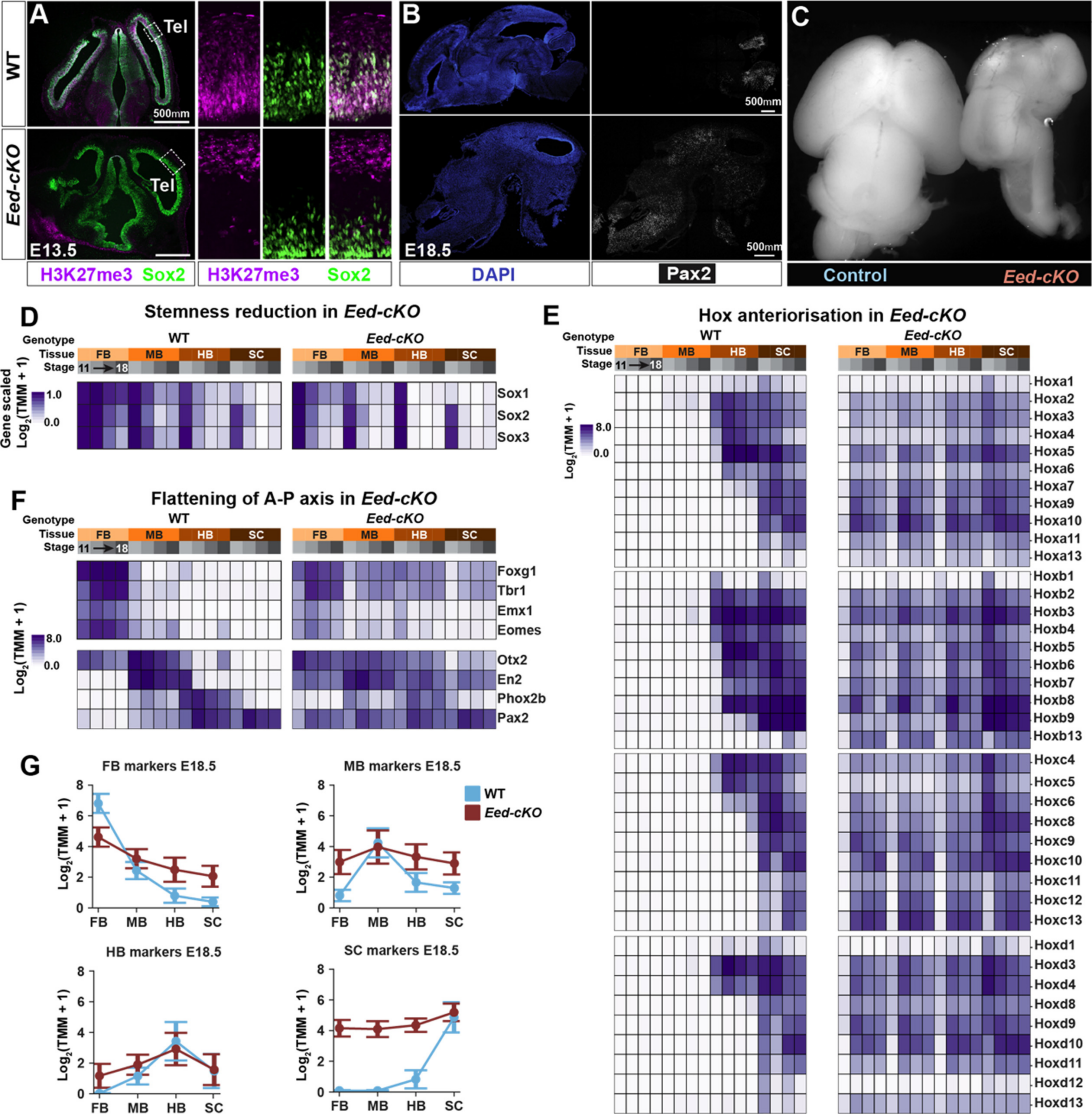
PRC2 gates the CNS A-P landscape. (**A**) Immunostaining for H3K27me3 and Sox2 (progenitors) in the WT and *Eed-cKO* telencephalon (Tel), at E13.5. At E13.5, deletion of *Eed*^*fl*/*fl*^ by *Sox1-Cre* results in loss of H3K27me3 from the CNS and staining is only observed outside of the CNS and in infiltrating blood vessels and blood cells. (**B**) Staining for DAPI (nuclei) and Pax2 in WT and *Eed-cKO* at E18.5 reveals ectopic expression of Pax2 in the entire FB and MB in the mutant. (**C**) WT and *Eed-cKO* littermate brains show undergrowth in the mutant. (**D**) In WT, there is a spatio-temporal gradient of progenitor gene expression (*Sox1/2/3*), i.e. a gradient of ‘stemness’ in the CNS, evident by prolonged expression of *Sox1/2/3* in the FB and MB. In *Eed-cKO*, the stemness phase in the FB and MB is shortened and the anterior CNS becomes more similar to the posterior region. (**E**) 35 of the 39 Hox homeotic genes show ectopic anterior expression in *Eed-cKO* mutants. (**F**) In WT, expression of spatial marker genes is restricted to specific A-P regions. In *Eed-cKO*, posterior genes (e.g. Pax2) are ectopically expressed in the brain, and anterior genes are reduced in the FB and MB and ectopically expressed in the HB and SC. The effects are less pronounced at E11.5, in line with the gradual loss of H3K27me3 at E10.5–E11.5. (**G**) A flattening of the expression is observed (mean and standard error) for each of the marker gene groups along the A–P axis, exemplified by trends in E18.5 (genes are listed in Supplementary Table S1).

To inactivate the PRC2 complex in the mouse CNS, *Sox1-Cre* was used to conditionally delete *Eed*^*fl*/*fl*^ in the CNS (denoted *Eed-cKO* herein). This resulted in the inactivation of *Eed* at E8.5, with a gradual reduction of the H3K27me3 mark, presumably due to replication-mediated dilution, until it is undetectable by immunostaining in the CNS proper, at E11.5 (5), visualized at E13.5 herein (Figure 1A). *Eed-cKO* embryos displayed a striking upregulation of posterior genes, such as *Pax2* (Figure 1B), in the FB/MB, and severe brain underdevelopment (Figure 1C), in large part due to a truncated proliferation phase (5).

We conducted a total of 64 RNA-seq experiments across wild type (*Eed*^*fl*/*fl*^; referred to as WT) and *Eed-cKO*, at four developmental stages E11.5, E13.5, E15.5 and E18.5, and of four tissues FB, MB, HB and SC. We opted to perform these experiments with bulk technology, enabling statistically robust assessment of genes associated with temporal and spatial specificity, which in turn, can guide future determination of cell type heterogeneity from single cell data (28).

Analysing the WT RNA-seq data for expression of the neural progenitor stemness genes *Sox1/2/3* (59) underscored the spatio-temporal stemness gradient (Figure 1D). In *Eed-cKO*, both the FB and MB displayed a more rapid downregulation of *Sox1/2/3*, while the HB and SC were less affected (Figure 1D). Analysis of spatially distinct TF markers (Supplementary Table S1) in WT revealed the expected selective gene expression along the A-P axis (Figure 1E, F). In contrast, in *Eed-cKO* mutants FB, MB and HB markers were downregulated in their specific regions, and ectopically upregulated in adjacent regions (Figure 1F, G). SC markers (e.g. 35 of the 39 Hox genes) were ectopically expressed in anterior regions (Figure 1E–G). The mutant effects were less pronounced at E11.5 (Figure 1E), in line with the gradual loss of the H3K27me3 mark during E10.5-E11.5 (5). These results revealed that *Eed-cKO* mutants displayed a striking ‘flattening’ of the CNS A–P axis, evident from the downregulation of brain TFs, the ectopic expression of Hox genes in the brain, and anterior downregulation of stemness genes.

### PRC2 inactivation results in posteriorization of the anterior CNS

**Figure 2.**
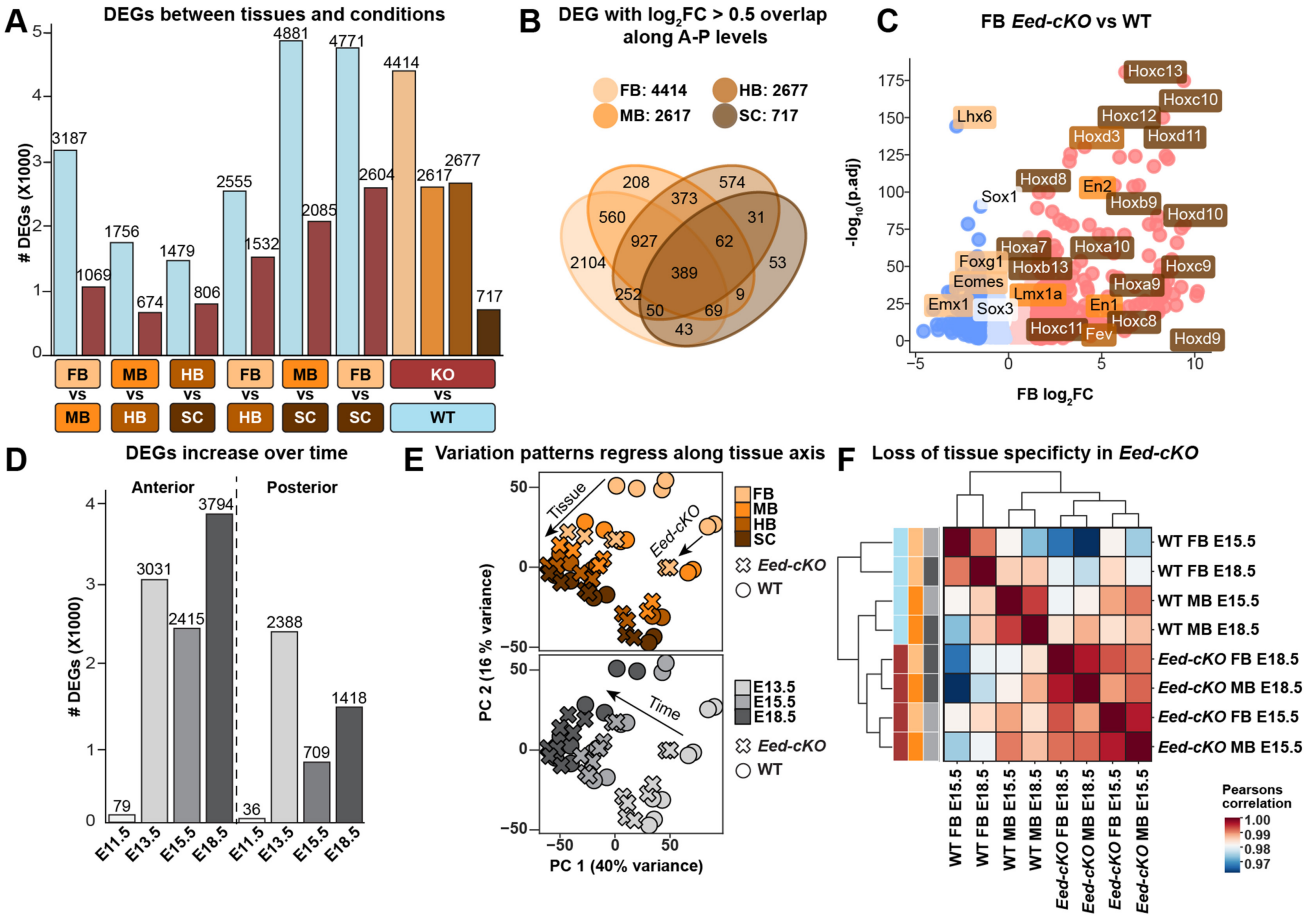
*Eed-cKO* mutation flattens the CNS A-P gradient. (**A**) Comparative DEG analysis between different CNS levels, in WT and *Eed-cKO*, based upon pooled time points. In WT, adjacent CNS tissues display fewer differences than distal ones, especially when compared to SC (DEGs; log_2_FC > 0.5, *P* < 0.05, where log_2_FC is the log_2_ transformed change). In *Eed-cKO*, the number of comparative DEGs are strongly reduced. *Eed-cKO* strongly affects anterior tissues while the SC is less affected. (**B**) Similar tissues (e.g. FB and MB) have a larger overlap between genes identified with a significant effect between *Eed-cKO* vs. WT. (**C**) *Eed-cKO* versus WT expression in FB shows that Hox genes are upregulated while brain-specific genes are downregulated. (**D**) DEG analysis over time for combined anterior (FB and MB) and posterior (HB and SC) tissues reveals that *Eed-cKO* affects the anterior more than the posterior CNS, and that the effects increase over time. (**E**) PCA of normalised RNA-seq count profiles labelled with tissue, condition (WT versus *Eed-cKO*) and time (E13.5–E18.5; E11.5 omitted); arrows indicate the A–P shift induced by *Eed* mutation. (**F**) Correlation between late stage anterior tissues showing a reduction of tissue specificity in *Eed-cKO*.

Analysing the global gene expression differences along the CNS A–P axis, we found major differences in the baseline WT transcriptomes, with FB and MB being strikingly different from the SC (Figure 2A). When compared to SC, the FB showed 4771 differentially expressed genes (DEGs) and MB 4881 DEGs (|log_2_FC| >0.5, *P* < 0.05, where log_2_FC is the log_2_ transformed fold change; pooled time points) (Figure 2A). In addition, all other comparisons revealed substantial gene expression differences, underscoring the uniqueness of each axial level (Figure 2A).

These axial differences were reduced in *Eed-cKO* mutants, with gene expression differences almost halved when comparing FB to SC, and MB to SC (Figure 2A). The FB was most affected, with 4414 DEGs, while SC displayed considerably smaller effects, with only 717 DEGs (Figure 2A, Supplementary Figures S1–S3). Surprisingly, only 389 genes were shared across all four tissue analyses, indicating that for the majority of DEGs the role of PRC2 is specific to each axial level (Figure 2B). While PRC2 inactivation generally caused upregulation (e.g. Hox genes), analysis of the FB revealed that a number of brain-specific TFs were downregulated (Figure 2C).

To investigate temporal variation throughout development, we grouped the FB and MB into ‘anterior’, and the HB and SC into ‘posterior’ sections. This revealed that the A-P axis differences between WT and *Eed-cKO* were most pronounced in the anterior CNS at E18.5 (Figure 2D). However, the largest increase in DEGs occurred between E11.5 and E13.5, in both the anterior and posterior tissues (Figure 2D).

PCA of normalised RNA-seq profiles (Figure 2E) indicated that *Eed-cKO* mutants are posteriorized along the A-P axis. Specifically, the mutant FB transcriptome was more similar to the WT MB, and the mutant MB to the WT HB. This trend was most evident at E13.5 but observed at all later stages (Figure 2E). The posteriorization of each *Eed-cKO* tissue was also evidenced by quantification of the normalised sum of square differences between the transcriptomes (Supplementary Table S2) and correlation between anterior samples (Figure 2F).

### PRC2 inactivation does not trigger extensive ectopic expression of non-CNS genes

To address if CNS-specific PRC2 inactivation resulted in ectopic expression of peripherally expressed genes, we surveyed for genes that were not expressed in the CNS at any axial level or stage but were activated in *Eed-cKO* mutants (mean TMM expression ≥ 0.5). Somewhat surprisingly, we only identified 213 genes in this category (Supplementary Figure S4, Supplementary Table S2). Hence, in contrast to the extensive A–P gene expression changes within the CNS, inactivation of PRC2 in the CNS did not result in widespread breakdown of germ layer barriers of gene expression (Supplementary Figure S4A–D).

### H3K27me3 only partly explains widespread effects of PRC2 inactivation

**Figure 3.**
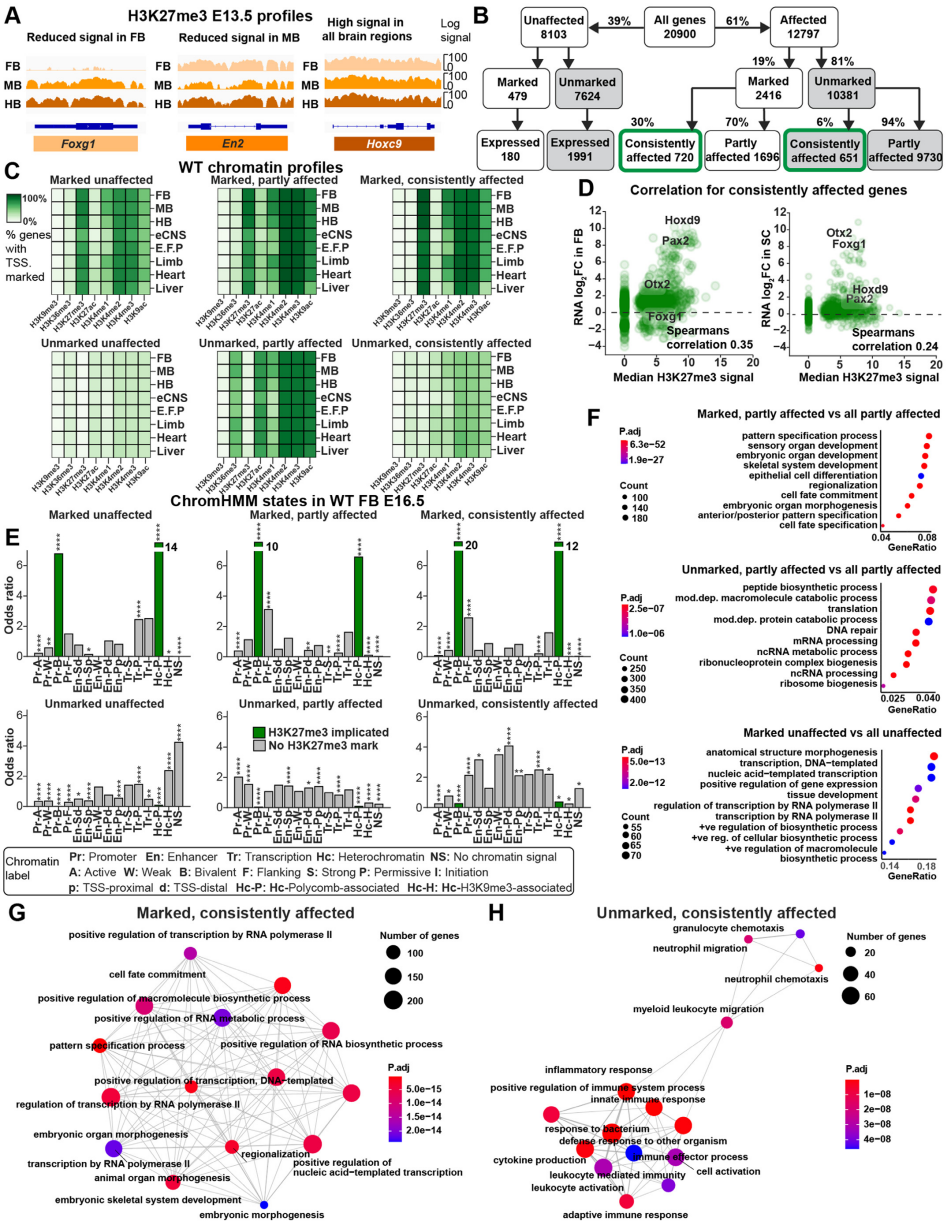
Direct and indirect control by PRC2. (**A**) H3K27me3 signal for three markers genes, at E13.5 in WT, showing a direct relation between H3K27me3 and expression (Figure 1E, F). (**B**) Data stratification based upon labels representing expression response to *Eed-cKO* mutation and the presence of H3K27me3. (**C**) Chromatin profiles in the CNS and reference tissues of genes stratified by expression changes and wild type H3K27me3 state. (**D**) FB log_2_FC and SC log_2_FC from consistently affected genes show minor correlation with the median H3K27me3 signal across brain tissues. (**E**) Enrichment of ChromHMM chromatin states, expressed as the odds ratio between positives in each gene category vs. all genes. (**F**) Functional Gene Ontology (GO) enrichment analysis of partly affected and unaffected genes shows greater similarity between marked groups irrespective of response to PRC2. (**G**) GO enrichment analysis of consistently affected and marked genes identifies terms related to embryonic development along the A–P axis. (**H**) GO enrichment of consistently affected and unmarked genes reveals ‘inflammatory response’ and ‘positive regulation of immune system process’ (edges represent shared genes between GO terms).

Publicly available WT H3K27me3 ChIP-seq data (46) revealed that the region- and gene-specific profiles for several of the spatially restricted genes, *Foxg1*, *En2* and *Hoxc9*, were consistent with a direct repressive role of the PRC2 complex (Figure 3A).

However, gene expression changes caused by PRC2 inactivation may result from layers of regulation when considered across the developmental trajectory. To begin addressing this issue in a systematic manner, we performed a ‘label-stratified’ analysis, sorting genes into categories based on gene expression response along with histone modification profiles. We labelled each gene as unaffected, partly affected or consistently affected, by using the expression response to PRC2 inactivation as per the DE analyses (Figure 3B). Thereby, the H3K27me3 state and expression response to *Eed-cKO* jointly defined six exclusive categories of genes (Figure 3B), see Materials and Methods for details.

Given PRC2’s role in maintaining tissue specificity, we hypothesised that genes in each category would display chromatin profiles that were specific to tissue. Within each gene category histone modifications in FB, MB, HB, neural crest (eCNS), embryonic facial prominence (EFP), limb, heart and liver, at E16.5 (46) were surprisingly similar, but between categories differences appeared (Figure 3C). Specifically, genes in the three H3K27me3-marked categories were commonly marked with H3K4me2/3 marks, but not H3K36me3, indicating their bivalent status (Figure 3C). Within the three H3K27me3-unmarked categories, active marks (H3K36me3, H3K27ac, H3K4me2/3) were primarily observed in the partly affected category (Figure 3C).

To further investigate the relationship between the H3K27me3 mark and gene expression, we computed the correlation between FB log_2_FC and H3K27me3 signal in the consistently affected gene category (Figure 3D). We found a limited positive correlation (ρ = 0.35, *P* < 0.01) between H3K27me3 and FB log_2_FC. This exceeded the correlation between the log_2_FC in all other tissues and the H3K27me3 signal, specifically SC log_2_FC: ρ = 0.24, and *P* < 0.01, (Figure 3D) HB log_2_FC: ρ = 0.08, *NS*, and MB log_2_FC: ρ = 0.24, *P* < 0.01 (Supplementary Figure S5A).

The limited correlation between H3K27me3 and gene expression changes prompted us to investigate whether a combination of histone marks i.e., chromatin states in a gene promoter could provide greater insight into the gene’s response to PRC2 inactivation. To this end, we assigned ChromHMM-predicted epigenetic states to each gene, based upon FB at E16.5 (18). We found that genes in all three H3K27me3-marked categories (unaffected, partly affected and consistently affected) exhibited similar epigenetic states, with strong signals for the Pr-B (promoter-bivalent) and Hc-P (heterochromatin-permissive) states (Figure 3E). The enrichment of these H3K27me3-implicated states was considerably higher for the consistently affected category, showing that if a gene is marked by H3K27me3 at E16.5 in the FB, it is likely to be affected by knocking out *Eed* (Figure 3E). The consistently affected unmarked genes stood out, with higher enrichment of enhancer states: En-Sd (Enhancer-Strong-TSS-distal), En-W (Enhancer-Weak-TSS-distal) and En-PD (enhancer-poised-TSS-distal) (Figure 3E), suggesting that these genes are indirectly regulated by PRC2. There were approximately equivalent numbers of genes in the consistently affected categories that were unmarked and marked (Figure 3B) indicating that lack of the H3K27me3 mark did not rule out effects in *Eed-cKO*.

To understand the functional heterogeneity of genes within and between each category, we also tested for over-represented Gene Ontology (GO) terms associated with their proteins (Figure 3F–H). For the H3K27me3-marked categories, both partly and consistently affected genes were enriched for A–P axis related terms, (e.g. pattern specification) (Figure 3F, G). Surprisingly, H3K27me3-marked but unaffected genes were also enriched for regulation and development, indicating that not all marked developmental genes were affected by *Eed-cKO* (Figure 3F). Unmarked and partly affected genes were associated with RNA processing terms (Figure 3F), while the unmarked, consistently affected genes were primarily enriched for immune response genes (Figure 3H).

### Variational Autoencoder finds latent codes for mixture of features

**Figure 4.**
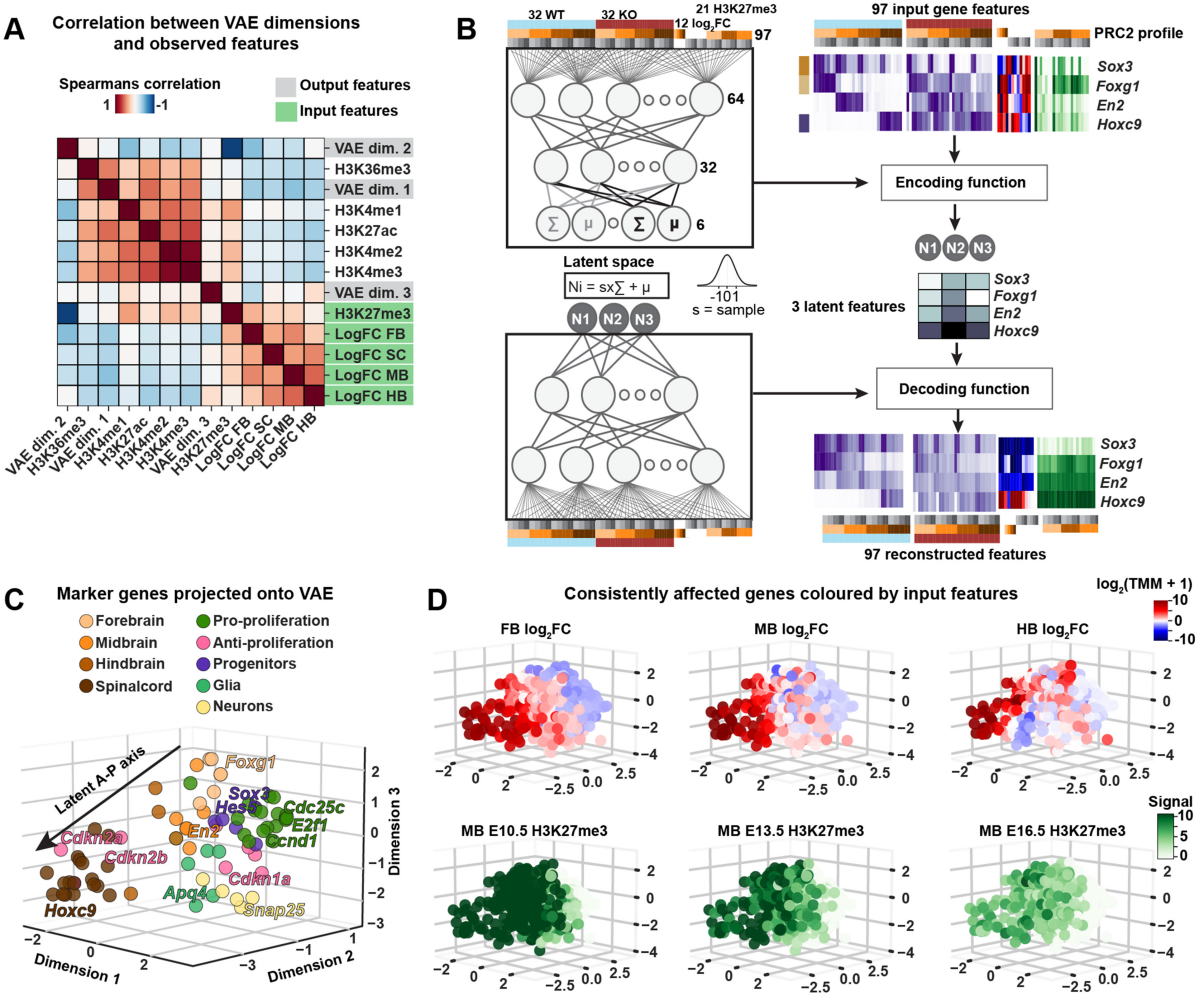
Integrated PRC2 profile forms latent A-P axis. (**A**) Spearman’s ρ across all consistently affected genes between VAE latent codes and selected observed summary features. (**B**) Simplified VAE model and the gene-specific input to the VAE, supplying a range of experimental observations (example genes shown) to a (non-linear, trainable) ‘encoding function’, which defines a latent code for each gene. A ‘decoding function’ is trained to reconstruct profiles for each gene, subject to VAE constraints. (**C**) Selected marker genes plotted in VAE *D* = 3 latent space, showing an A–P gradient. (**D**) Consistently affected genes plotted in VAE latent space, coloured (top row) by log_2_FC *Eed-cKO* versus WT in FB, MB and HB, and (bottom row) the median signal in H3K27me3 across developmental time points.

Genes marked by H3K27me3 in the CNS tended to be affected by *Eed-cKO*. However, the effect on unmarked genes was nebulous e.g., correlating the log_2_FC of the response with the experiment-wide median H3K27me3 state revealed only a weak correlation (FB: ρ = 0.35, *P* < 0.01, SC: ρ = 0.24, *P* < 0.01), underscoring the limited ability of the H3K27me3 mark alone to predict the expression response to *Eed-cKO* (Figure 4A). These findings suggested that labelling genes without jointly including details of developmental stage and tissue obscured features required to identify co-regulated genes.

We defined a PRC2 profile of prioritised features (97) representing each gene for input to the VAE: RNA-seq data for WT and *Eed-cKO*, log_2_FC from the DE analyses, and WT H3K27me3 signal. Our goal was to integrate the data into a relatively small set of features and use the model to interrogate relationships between the WT H3K27me3 signal and the gene expression response to *Eed-cKO* (Figure 4B).

A VAE was trained on PRC2 profiles, to find a latent ‘code’ for each gene (Figure 4B). Because the results were reproducible and robust to parameter perturbations, the VAE architecture and parameters were chosen with minimal tuning (see Materials and Methods). When the VAE used three or more hidden nodes, referred to as latent dimensions (*D*), we observed only minor reconstruction loss (Supplementary Figure S6). Hence, at *D* ≥ 3, intermediate layers captured sufficient information to successfully decode essential variation across the full data set. We tested two versions of the data set: the 1371 consistently affected genes (as defined above), and all of the 12 797 affected genes, with *D* = 3 and *D* = 6. Based upon these findings, we subsequently used the VAE with *D* = 3, trained with the consistently affected gene data set.

### VAE latent code places genes along A–P axis

While no pair of VAE dimensions correlated measurably (|ρ| < 0.1 for all pairs), as anticipated, the VAE dimensions correlated with a number of input features (Figure 4A). For instance, dimension 2 correlated negatively with the median H3K27me3 signal (ρ = −0.93, *P* < 0.01) and mildly with FB log_2_FC (ρ = −0.42, *P* < 0.01).

To further validate that the VAE latent code uncovered biologically relevant CNS features, we tracked the aforementioned marker genes (Supplementary Table S1). We noted that the FB, MB, HB and SC genes were placed along a latent version of the A-P axis (Figure 4C). In addition, investigating the placement of proliferation genes (Supplementary Table S1) we noticed that pro-proliferative genes were placed in the vicinity of FB genes, while two well known anti-proliferative genes (*Cdkn2a* and *Cdkn2b*) were placed adjacent to the SC genes, in agreement with the enhanced anterior proliferation (Figure 4C). Markers for neurons and glia did not group along the latent A–P axis, rather projecting onto a distinct segment orthogonal to the axis, in line with the generation of these cell types at all axial levels during the embryonic stages analysed (Figure 4C).

The VAE latent space also captured several other key features of PRC2 control of the developing CNS. These included the graded involvement of PRC2 along the A-P axis, with extensive gene upregulation in the *Eed-cKO* FB and smaller effects in the HB (Figure 4D). We also observed a temporal reduction in the H3K27me3 mark, as evident in the MB from E10.5 to E16.5 (Figure 4D).

### VAE latent dimensions identify co-regulated but functionally diverse genes

**Figure 5.**
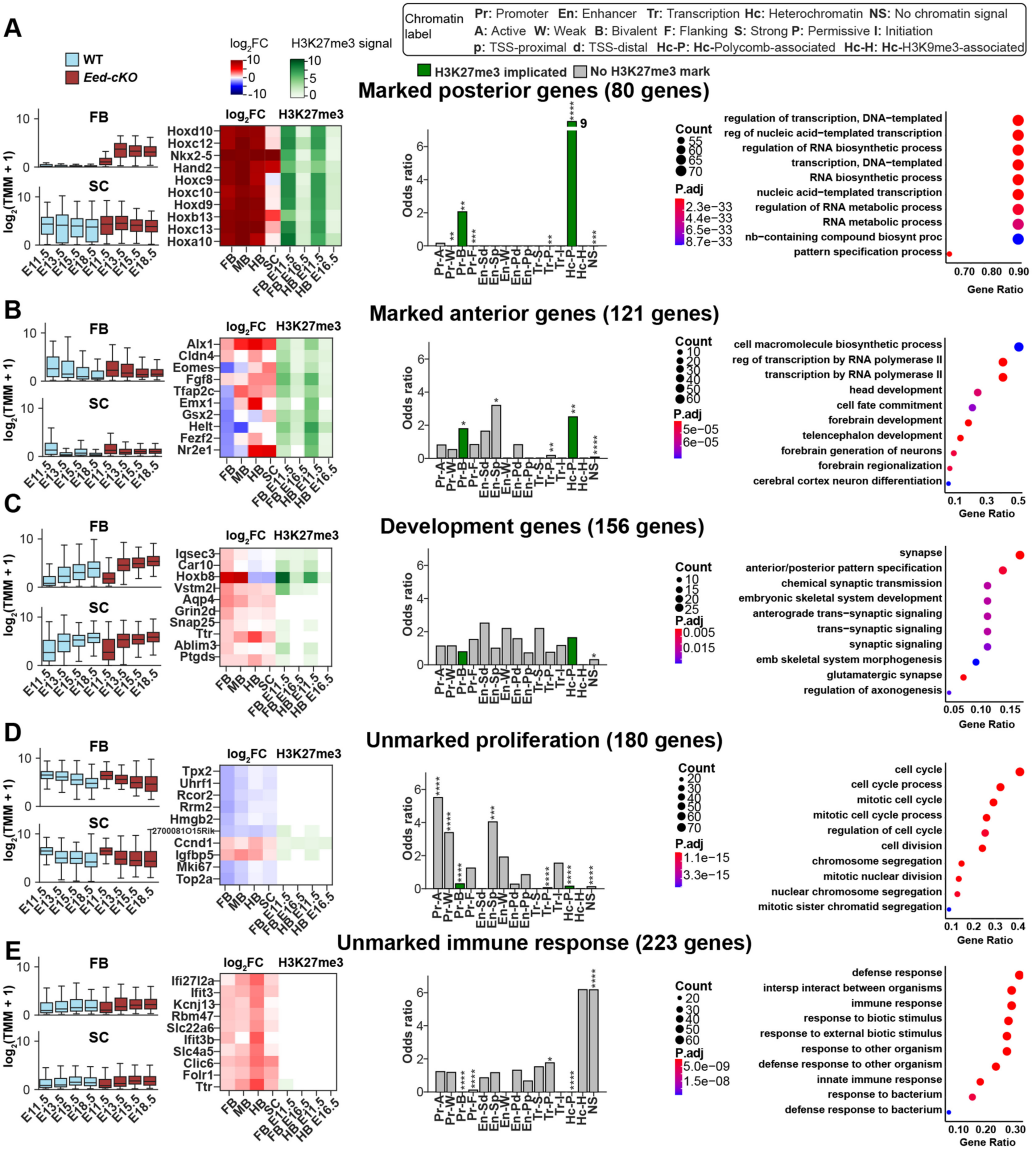
VAE identifies functionally diverse gene cohorts. (A–E) Different cohorts of genes identified at tails of one or more VAE latent dimensions (±1.25 SD from mean). (**A**) In the WT, the marked posterior gene cohort is repressed in the FB and expressed in the SC, while in *Eed-cKO* mutants, they are overexpressed in FB, while the SC is unaffected. Top-10 genes in this group are predominantly Hox genes, marked by H3K27me3 in both FB and HB. Enrichment of ChromHMM chromatin states and GO terms show bivalent and repressive states, and enriched gene regulation terms, respectively. (**B**) In WT, the marked anterior gene cohort is repressed in SC and expressed in FB. In the *Eed-cKO* mutants, they are mostly downregulated in FB and upregulated in SC. Top-10 genes display tissue specific response to *Eed-cKO*. Marked anterior genes show minimal enrichment of specific chromatin states. FB differentiation and development GO terms are enriched. (**C**) The development gene cohort increases in expression over time and is more highly expressed in SC than FB, a trend more pronounced in the mutant. Top-10 genes display upregulation in the mutant. There is no enrichment of specific chromatin states. Embryonic development associated GO terms are enriched. (**D**) The unmarked proliferation gene cohort decreases in expression over time, in both the WT and *Eed-cKO*, which is pronounced in *Eed-cKO*. Enriching for active and weak promoter chromatin states and GO cell cycle functions, suggesting that active genes are important for cell growth and the rate of proliferation. (**E**) The unmarked immune response gene cohort displays no specific expression profile in WT, but are upregulated in the mutant. Top-10 genes show a homogenous response to *Eed-cKO*, in particular a strong upregulation in HB. These genes are enriched for the no signal (NS) ChromHMM chromatin state and immune response GO terms.

The variation captured by the VAE enabled the discovery of new cohorts of genes that coincided with each latent dimension. We grouped genes by their existence at the extremes of each dimension, with membership determined by being in the tail of the distribution (±1.25SD from mean), resulting in six non-exclusive cohorts of genes. One cohort was omitted from further analysis, as it contained genes that extensively overlapped with the other five cohorts (Supplementary Figure S13A). For the five remaining cohorts we used GO term enrichment and gene expression patterns to manually label the cohorts, yielding: (i) posterior genes, (ii) anterior genes, (iii) development genes, (iv) unmarked proliferation genes and (v) immune response genes (Figure 5A–E). The identification of immune response genes was unanticipated, because *Sox1-Cre* was previously found to not delete *Eed* in the blood vessels and blood cells (5). To probe the underpinnings of this effect we stained for IB4, which revealed a higher ratio of blood cells and blood vessels in comparison to the CNS tissue in the *Eed-cKO* (Supplementary Figure S14).

The posterior gene cohort was repressed in the FB and MB in WT and upregulated in *Eed-cKO* (Figure 5A). This cohort was enriched for ChromHMM bivalent promoter states, suggesting that these genes are directly controlled by PRC2 and are selectively expressed (Figure 5A). The anterior gene cohort tended to exhibit an opposing RNA expression profile to the posterior genes, with a decrease in expression over time, and limited enrichment of H3K27me3-associated chromatin states (Figure 5B). The development cohort included a mixture of genes that were mostly upregulated in *Eed-cKO*, and whose ChromHMM profile indicated both direct and indirect PRC2 effects (Figure 5C). The unmarked proliferation cohort was enriched for cell cycle genes, mostly those with pro-proliferative function (Figure 5D). In WT, genes in this cohort displayed a logical downregulation as neurogenesis comes to an end in both FB and SC. Relative to WT, PRC2 inactivation accelerated the decrease in expression of this cohort in all tissues, but most distinctly in FB (Figure 5D). Lastly, the immune response cohort was weakly upregulated in *Eed-cKO* and enriched for the absence of PRC2-associated ChromHMM marks, indicating an indirect effect of PRC2 (Figure 5E).

To address if the WT profile of gene cohorts were conserved in humans, in particular regarding the expression of the genes in the marked anterior cohort, we analysed publicly available data from PsychEncode (58). We found similar expression patterns across the mouse and human orthologs, with the marked anterior genes displaying significant tissue specific effects in human embryonic brains (Supplementary Figure S15A).

### The recovery of relevant gene groups is method dependent

VAE allows low-dimensional codes to capture non-linear relationships from a high-dimensional input space, staggered at each intermediate layer (60). The layout of the latent codes reveals genes that share systematic and relevant expression changes and repressive states across time points and space, PRC2 perturbed or not.

To quantify the extent and nature of organisation of the VAE relative to representative dimensionality reduction methods, including PCA, UMAP, tSNE and PHATE, we performed a number of tests.

First, we asked if each method at *D* = 3 and at *D* = 6 had the capacity to find latent codes that distinguished genes with known but different A–P association (defined by Gene Ontology, see Materials and Methods) in both the consistently affected and affected datasets. Using the Silhouette score as a measure for set separability, we found that VAEs outperformed the other methods in the majority of cases (Supplementary Figure S7A, B). However, the low Silhouette scores highlight the inherent difficulty in separating the gene sets, which are distinguished only by their association with the A–P axis in development.

Second, we asked whether the groups that were formed by each method displayed similar biological enrichment. This test involved for each method selecting genes at the tails of each component at *D* = 3; these gene groups were then evaluated for enrichment in GO terms and biological pathways. All methods were able to distinguish the most salient functional groups, e.g., the posterior group. However, only the VAE and tSNE were able to distinguish an anterior gene cohort (Figure 5B, Supplementary Figures S8 and S9). PCA and UMAP had duplicate groups relevant to cell cycle (Supplementary Figure S10 and S11), while PHATE uniquely identified a group containing ‘membrane’ and ‘signalling’ terms (Supplementary Figure S12). The gene cohorts we defined using the VAE analysis were broadly captured by tSNE, which in turn showed to be less capable of separating GO-defined sets (Supplementary Figure S7B).

### PRC2 regulates cell cycle genes directly or by proxy TFs

**Figure 6.**
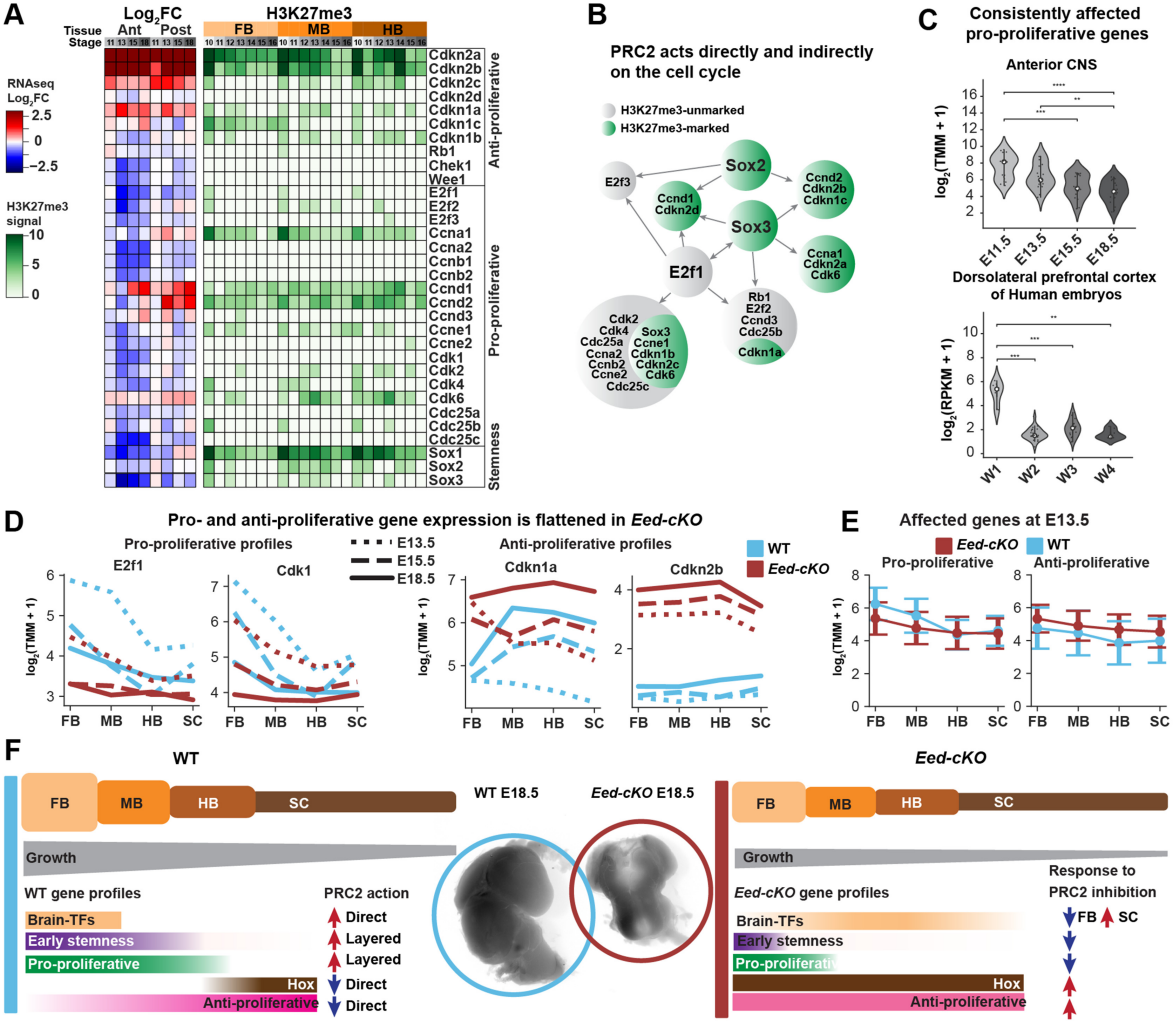
Layered cell control by PRC2. (**A**) Cell cycle gene response to PRC2 inactivation shows strong upregulation of some marked and consistently affected anti-proliferative genes, while the majority of pro-proliferative genes are downregulated and unmarked. Sox genes are marked and downregulated. (**B**) Proposed mechanism of action for the indirect regulation of the Sox genes on the cell cycle genes. (**C**) Consistently affected pro-proliferative genes (E2f1, Ccna2, Ccnb1, Cdc25c and Ccnd1) exhibit a reduction in expression over embryonic development. This trend is observed in mice across the anterior brain regions (FB and MB), and in human embryonic samples (human homologs: ENSG00000134057/CCND1, ENSG00000101412/E2F1, ENSG00000145386/CCNA2, ENSG00000110092/CCND1) (W1: 8–9 postconceptional weeks (PCW), W2: 12–13 PCW, W3: 14–18 PCW, W4: 18–22 PCW) (58), also across the brain (dorsolateral prefrontal cortex). (**D**) Select cell cycle genes exhibit evidence of an A–P gradient in WT. (**E**) Grouping all affected cell cycle genes reveals a trend (mean and standard error) for A–P flattening. (**F**) PRC2 ensures that Hox homeotic genes are only expressed in the SC and HB, and brain TFs only in the FB and MB, and promotes gradients of stemness, anti- and pro-proliferative gene expression. These A–P differences in regulatory gene expression underpin A–P differences in proliferation, creating a gradient of CNS growth

A key phenotype of *Eed-cKO* mutants is a striking reduction of proliferation of the FB (Figure 1C) (5). Moreover, the VAE analysis identified many genes (180 genes), in the ‘unmarked proliferation’ cohort (Figure 5D). These findings prompted us to focus on the expression of the main cell cycle genes (32 genes; Supplementary Table S1), to explore the regulation of pro- and anti-proliferative genes (see Supplementary Figure S15B for workflow). Looking first at WT, we observed that the majority of pro-proliferative and anti-proliferative genes were expressed in opposing gradients along the A–P axis (Figure 6A, D). In *Eed-cKO* mutants, we found that the majority of cell cycle genes (29/32) were affected, with eight consistently affected and 21 partly affected (Figure 6A, Supplementary Table S1). With few exceptions, pro-proliferative genes were downregulated while anti-proliferative genes were upregulated relative to WT, and these effects were most pronounced in the anterior CNS (FB and MB, Supplementary Figure S15C), resulting in a general flattening of the gene expression gradients (Figure 6A, D, E). Two outliers were the pro-proliferative genes *Ccnd1/2*, which were strongly upregulated in the posterior CNS (HB and SC) (Figure 6A). The H3K27me3 profiles of the anti-proliferative genes were comparatively pronounced, although several pro-proliferative genes, such as *Ccnd1/2* and *Ccna1*, were also marked (Figure 6A).

Because the majority of pro-proliferative genes appeared to be indirectly affected by PRC2, we sought to identify which TFs could be targeting the proliferation genes. We again focused on the *Sox1/2/3* stemness genes, as well as E2f1, a core TF in the cell cycle machinery (61). *Sox1/2/3* were marked by H3K27me3, while *E2f1* showed limited marks, if any (Figure 6A). However, all four genes were downregulated in *Eed-cKO* (Figure 6A). Previous ChIP-seq studies have probed the genome-wide occupancy of three of these four TFs (62–64). These data revealed that Sox2 and -3 bind to a number of proliferation genes, including E2f1 and other E2f genes, and that E2f1 binding showed extensive overlap with the Sox2/3 binding profiles (Figure 6B). These findings suggest that PRC2 action is layered – acting both directly and indirectly, via *Sox1/2/3* and *E2f1/2/3*, to control cell cycle gene expression.

To investigate whether the cell cycle gene expression profiles are evolutionarily conserved, we tested whether the WT profile of early activation of consistently affected pro-proliferative genes is conserved in humans. We again used the publicly available data from PsychEncode (58) and confirmed a significant reduction over time in the pro-proliferative genes in human embryonic brain development (Figure 6C).

## DISCUSSION

### PRC2 promotes the developing CNS A–P axis

The developing CNS displays evolutionarily conserved patterning along the A–P axis, evident by the selective expression of brain-specific TFs anteriorly and Hox homeotic genes posteriorly (65,66). Studies in *Drosophila* have also revealed an A–P expression gradient of neural stemness genes (2). In *Drosophila*, the selective expression of brain-TFs, Hox genes and neural stemness genes is accompanied by and (to a great extent) drives gradients in pro- and antiproliferative gene expression, which in turn results in faster cell cycles, and the expansion of the anterior CNS (2–5). Studies in mouse have indicated that many of these developmental features are conserved in mammals, although the degree of conservation is unclear (5). Moreover, while PRC2 plays a key role in promoting these A–P differences, its precise roles have hitherto not been comprehensively addressed in mammals.

We observed striking gene expression gradients of stemness, pro- and anti-proliferative genes, demonstrating that these features are also conserved from *Drosophila* to mouse. We found that PRC2 inactivation resulted in extensive gene expression changes in the CNS. Looking specifically at the aforementioned developmental genes we found that PRC2 inactivation reduced brain-TF expression and upregulated Hox genes anteriorly. The gene expression gradient of stemness and pro-proliferative genes appeared flattened, and there was an upregulation of anti-proliferative genes. Hence, PRC2 plays a fundamental role in promoting anterior CNS development, with anterior tissues posteriorizing and reducing their stemness in *Eed-cKO* mutants (Figure 6F). These regulatory effects generally accumulate over time, i.e. once a gene becomes dysregulated it remains so.

### PRC2 inactivation causes extensive direct and indirect effects

Comprehensive analyses of H3K27me3 suggest that PRC2 plays a significant role in development and disease (67). However, metrics based on repressive marks alone are unable to explain the effects on the substantial number of unmarked genes. To understand the extent PRC2 acts in a direct or indirect manner upon the affected genes, we merged our 64 transcriptomes with relevant histone modification profiles. We then stratified the data based on cut-offs, sub-setting genes into six categories.

All partly/consistently affected gene categories with H3K27me3 were enriched for GO terms related to embryonic patterning, which aligns well with the observed effect of *Eed* mutation i.e., a flattening of the CNS A-P axis. This finding, combined with their ChromHMM states, indicates that this gene group is directly regulated by PRC2. The H3K27me3-marked and unaffected gene category was enriched for similar GO terms, i.e. regulation and development, showing that a subset of H3K27me3-marked developmental genes are not affected by *Eed-cKO*.

There were many partly/consistently affected genes without H3K27me3, suggesting that the indirect effect of PRC2 inactivation is both comprehensive and diverse.

### VAEs distinguish gene cohorts relevant to A-P axis control

While we could have extended our label stratified analysis to include other factors, e.g. ‘up’ or ‘down’ in each DE analysis, the number of gene categories increases exponentially. In contrast, using a dimensionality reduction method, such as the VAE, constrains our comparisons while minimising the information loss. The VAE was able to distinguish between genes with qualitatively different functional profiles and multi-variate trends across the datasets. Moreover, several genes within each cohort were surprisingly varied in terms of both expression changes and chromatin state, indicating that the multi-variate nature of the VAE analysis uncovers a spectrum of biologically relevant, gene groupings across gene expression and histone modification features.

Recent single cell annotation methods have highlighted the utility of incorporating ‘gene sets’ to guide cell type identification, (27), while others emphasise the importance of feature selection to improve analyses (28,68). Using VAEs and multi-omic bulk-data we are able to robustly identify co-regulated genes relevant to A-P axis control, suggesting gene cohorts that could guide the recovery of key cell types in time and space, even in inherently sparse single cell data, such as multi-omic single cell assays (69).

### Immune response genes may be affected by several mechanisms

Unexpectedly, immune response genes were identified as a salient function affected by PRC2. Using *Sox1-Cre* to delete *Eed* only removes gene function in the CNS itself, and not in the blood cells or blood vessels (Supplementary Figure S14; (5)). It is therefore possible that the increased expression of immune response genes in the undergrown FB and MB in *Eed-cKO* mutants may simply result from a higher ratio of blood vessels/immune cells to CNS cells, thereby increasing the transcriptome signal in an indirect manner. Indeed, our finding of increased IB4 staining, a marker for microglia and blood vessels, in relation to both the Sox2 and DAPI staining supports this notion (Supplementary Figure S14). However, two other plausible causes of activation of immune response genes are (I) a CNS-autonomous effect, as PRC2 has been linked to immune responses in human cancer (70) and/or (II) that the developmental defects in *Eed-cKO* mutants lead to a breakdown of the blood brain barrier and/or an immune response to a malforming CNS. Indeed, our previous studies did reveal that apoptosis occurred at earlier stages in the *Eed-cKO* CNS (5). The latter explanation would also be supported by the increased IB4 staining. Further studies i.e., spatio-temporal single cell RNA-seq, would be required to determine why the immune response genes are activated.

### Layered control of proliferation by PRC2

Cell cycle genes (Supplementary Table S1) were scattered across marked and unmarked, and differently affected categories obscuring the biological signal in the label stratified analysis. In contrast, VAE analysis highlighted consistently affected pro-proliferative genes in a proliferation cohort, suggesting a common regulatory regimen, and that we probe the regulation of cell-cycle genes further. In general, pro-proliferative genes were downregulated and anti-proliferative genes upregulated, and there was a general flattening of their A–P expression gradients. These gene expression changes are likely directly responsible for the undergrowth phenotype observed in the mutant FB and MB. Analysis of the H3K27me3 profiles revealed that PRC2 may be acting directly on a subset of marked proliferation genes, and likely indirectly, via e.g. the Sox1/2/3 and E2f1/2/3 TFs, on unmarked proliferation genes.

The tendency for PRC2 to directly regulate anti-proliferative genes and indirectly regulate pro-proliferative genes, points to an uneven involvement of the epigenetic machinery in cell cycle regulation. This finding is not surprising given the different evolutionary origin of the cell cycle genes and the gradual emergence of the epigenetic machinery. Specifically, while the basic core cassette of Cyclins and Cdks is ancient in eukaryotes (71) the Kip/Cip family evolved later, and INK4 even more recently (the INK4 family is not present in *Drosophila*). The Kip/Cip and INK4 families likely evolved to provide the increasingly refined control of proliferation necessary in larger metazoans. Indeed, evolution of the cell cycle machinery has gone hand in hand with, and one may argue been facilitated by, an increasingly elaborate epigenetic machinery. Against this backdrop, it is logical that PRC2 is heavily engaged in directly regulating the Cip/Kip and INK4 families, but indirectly regulating the more ancient cell cycle genes.

### PRC2 gates an ancient CNS stemness gradient

One of the key features of the developing CNS A-P axis is a stemness gradient, which drives CNS anterior expansion. PRC2 plays five key roles herein: (i) promoting brain-specific TF expression, (ii) repressing anterior Hox gene expression, (iii) promoting a gradient of neural stemness TF expression, (iv) repressing anterior anti-proliferative gene expression and (v) promoting anterior pro-proliferative genes (1). Our findings herein suggest that (i) PRC2 regulates the first four categories directly by application of H3K27me3 and (ii) PRC2 regulates pro-proliferative genes by also relying on proxy TFs.

Our spatio-temporal transcriptomic and epigenomic analysis provides an in-depth view into the strikingly different regulatory landscape present in the anterior versus posterior regions of the CNS, and the profound importance of PRC2 in establishing and driving these differences. Previous studies show that the role of PRC2 in gating A–P gene expression is integral for mouse, fly, and zebrafish development (8,72–73). Our work extends upon this, revealing that the FB genes dysregulated in the developing mouse PRC2 mutant CNS are also selectively expressed during human embryonic brain development, underscoring the evolutionary conservation of brain development across bilateria.

A number of observations in different species, based on gene expression analyses with anatomical and phylogenetic considerations, have led to the proposal that the anterior and posterior CNS may have originated from different parts of the nervous system present in the bilaterian ancestor, the apical and basal nervous systems (65,74–76). If true, this brain-nerve cord ‘fusion’ concept may help explain the strikingly different gene expression and neurogenesis properties of the brain, when compared to the nerve cord, as well as the apparent ‘brain-preoccupation’ of the PRC2 complex.

## DATA AVAILABILITY

Raw RNA-seq files are available at the NCBI/Gene Expression Omnibus under the accession GSE123331. Processed data and code including Jupyter Notebooks (both as HTML and ipynb) used to generate all results are available at: https://arianemora.github.io/mouseCNS_vae/.

We developed an interactive web site to inspect latent representations of genes: http://bioinf.scmb.uq.edu.au:81/cnsvae/static/.

Our analysis workflow has been packaged and documented to be widely applicable. In particular, the workflow is amenable to any biological system where a gene ‘profile’ is indicative of function and mode of regulation. We provide example code to use VAEs in R and Python.

## Supplementary Material

gkac006_Supplemental_File
