# Supplementary material for: Variational autoencoding of gene landscapes during mouse CNS development uncovers layered roles of Polycomb Repressor Complex 2
Source: Nucleic Acids Res. 2022 Jan 20;50(3):1280–96. doi: 10.1093/nar/gkac006 (PMC8860581; doi:10.1093/nar/gkac006)
Supplement: gkac006_Supplemental_File [file gkac006_supplemental_file.pdf]

SUPPLEMENTAL INFORMATION

Supplemental Table 1. Gene sets used throughout the paper. Bolded genes were used for display in Figure 4. Display genes were chosen based upon published gene expression patterns. Ectopic genes were identified computationally and indicates that the gene had a mean expression less than 0.5 TMM in the WT and greater than 0.5 TMM in the mutant.

Gene group	Gene names
Anti-proliferation (all)	**Cdkn1a, Cdkn2a, Cdkn2b**, Cdkn2c, Cdkn1b, Cdkn1c, Cdkn2d, Rb1, Check1, Wee1
Proliferation genes (all)	**Ccna1, Ccnd1, Cdc25a, E2f1, Mcm10, Cip2a**, Ccna2, Ccnb1, Ccnb2, Ccnd2, Ccnd3, Ccne1, Ccne2, Cdc25b, Cdc25c, E2f2, E2f3, Mcm5, Mcm3, Mcm2, Cdk1, Cdk2, Cdk4, Cdk6
SC genes	**Hoxb13, Hoxb9, Hoxb8, Hoxc13, Hoxc12, Hoxc11, Hoxc10, Hoxc9, Hoxc8, Hoxd11, Hoxd10, Hoxd9, Hoxa7, Hoxa9, Hoxa10,** Hoxb7, Hoxb6, Hoxb5, Hoxd13, Hoxd8, Hoxa6, Hoxa11, Hoxa13
HB genes	**Phox2b, Krox20, Fev, Hoxb1, Hoxd3**
MB genes	**En1, En2, Lmx1a, Bhlhe23, Sall4**
FB genes	**Emx1, Eomes, Tbr1, Foxg1, Lhx6**
Progenitors	**Sox2, Sox1, Sox3, Hes1, Hes5**
Neurons	**Snap25, Syt1, Slc32a1, Slc17a6, Syn1**
Glia	**Aqp4, Cspg4, Slc6a11, Olig1, Igfbp3**
Ectopic unmarked	Gsta3, Rfx8, Aox3, Mdh1b, Mogat1, Sp100, Ugt1a7c, Ugt1a6a, Iqca, Cfap221, Lax1, Fcgr4, Cd48, Vsig8, Ccdc170, Vip, Zc3h12d, Lilrb4a, Oit3, Aire, Lif, Ifi47, Slc36a2, Nmur2, Slc35g3, Mgl2, Clec10a, Tm4sf5, Ccl2, Ccl12, Slfn8, Krt26, Asb16, Aanat, Card14, Cbr2, Cdhr3, Efcab10, Acot4, Batf, Gpr65, Ifi27l2a, Serpina3g, Omd, Fam81b, Bhmt, Il31ra, Dhrs2, Arl11, Rubcnl, Epsti1, Slc45a2, Gpr20, Lypd2, Meltf, Cd80, Cd200r1, Btla, Wdr27, Tff3, H2-DMb1, H2-Eb2, C4b, Ly6g6d, H2-T22, Ankrd66, Guca1a, Plin4, Psma8, Zfp474, Iigp1, Ifit3b, Ifit1, Spaca9, Morn5, Spo11, Tnfsf10, Sptssb, Chia1, Ubl4b, 1700013F07Rik, Bank1, Cyp4b1, Rhbdl2, Pla2g5, Ccdc27, Slc26a5, Tlr6, Rhoh, Ppef2, Fam47e, Gbp11, Selplg, Cfap73, Lrrc43, Ccl24, Muc3a, Card11, Clec5a, Reg3b, Cacna2d4, Clec4a3, Clec4n, C1rl, C1ra, Fgf23, BC035044, Lmntd1, Apoc1, Capn12, Sec1, Isg20, Wdr93, Cfap161, Olfr558, Trim34b, F10, Gdf15, Abcc12, Dnaaf1, Kcng4, Casp12, 1700012B09Rik, Bcl2a1b, Gadl1, Hhatl, Ccr2, Akap14, Xlr, Dmrtc1a, Cysltr1, Tex16, Lhfpl1, Ace2
Ectopic marked	Tcf24, Cryba2, Ihh, Fcrlb, Tcf21, Nodal, Npffr1, Fstl3, Gipc3, Hand1, Alox12b, Hnf1b, Gcgr, Tc2n, Gsc, Lbhd2, Prss16, Susd3, Dmgdh, Ltb4r2, Gja3, Gata4, Fam83f, Ttll8, Wnt10b, Gsc2, Ildr1, Prss41, Hs3st6, Gng13, Nkx2-5, Mpig6b, Lta, Abcg8, Dmrt1, Acbd7, Spag6, Lrrc26, Cutal, Rspo4, Rem1, Fgf2, Hapln2, Rhbg, Slc44a3, Foxe1, Cdkn2a, Kdf1, Lrrc38, Gabrd, Cwh43, Nmu, Cfap299, Gfi1, Tbx5, Hoxa13, Vax2, Zfp541, Ppm1n, Phldb3, Lypd3, Nccrp1, Slc6a16, AC151602.1, Ano9, Ascl2, Rab20, Htra4, Adrb3, Hand2, Comp, Ttc29, Il15, Mlkl, 4833427G06Rik, C2cd4a, Ankrd34c, Prss50, Ccr9

Supplemental Table 2. Normalised distance between tissues. For each *Eed-cKO* condition the distance (normalised sum of square differences between gene expression) between the mutant's merged replicates and all WT conditions is shown. The WT condition with the smallest distance is to the *Eed-cKO* tissue is shown. There is an evident regression along the A-P axis of *Eed-cKO* FB samples, and some *Eed-cKO* MB samples being most similar to more posterior WT tissues (highlighted in bold).

Eed-cKO	**Most similar WT condition**	**Distance**
**ko11fb**	**wt11mb**	32.689
**ko13fb**	**wt13mb**	67.778
**ko15fb**	**wt15mb**	60.334
**ko18fb**	**wt18hb**	66.041
**ko11mb**	**wt11hb**	29.972
**ko13mb**	**wt13sc**	56.335
ko15mb	wt15mb	54.135
**ko18mb**	**wt18hb**	57.589
ko11hb	wt11hb	36.108
ko13hb	wt13sc	58.831
ko15hb	wt18hb	60.739
ko18hb	wt18hb	54.093
ko11sc	wt11sc	24.458
ko13sc	wt13sc	37.104
ko15sc	wt18sc	51.529
ko18sc	wt18sc	39.241

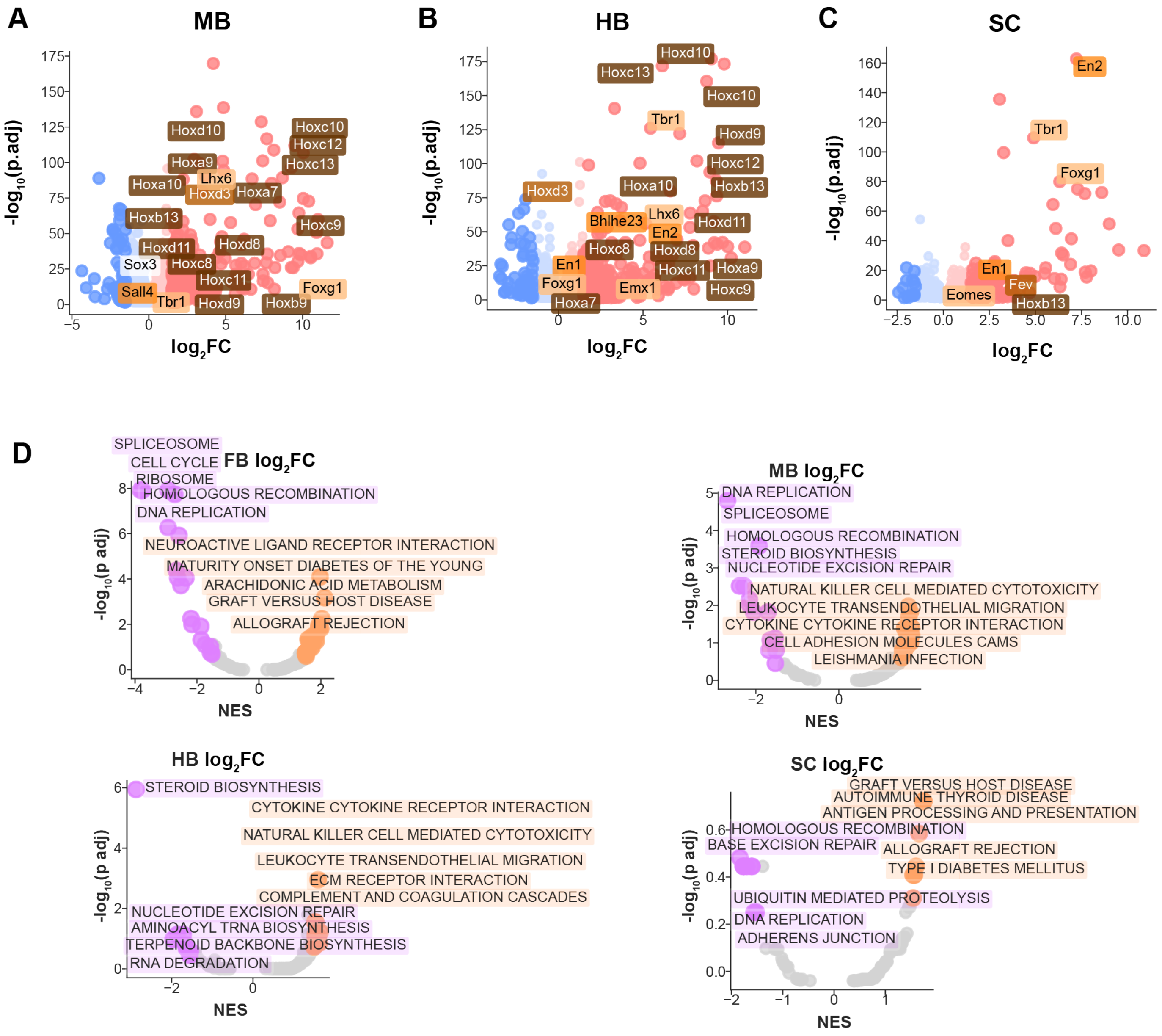

Supplemental Fig 1. Tissue specific response to *Eed-cKO* (A) In MB Hox genes are upregulated in *Eed-cKO* for E13.5 – E18.5. (B) In HB Hox genes and select FB genes, such as *Tbr1*, are upregulated in *Eed-cKO*. (C) In SC FB specific genes upregulated in *Eed-cKO*, with an overall smaller response than in MB and HB. (D) The $\log_2$FC for each tissue was used to rank the significant genes for GSEA showing that in FB and MB there is negative enrichment for the cell cycle pathway and RNA biology pathways, such as "ribosome" and "spliceosome" (these do not appear to be negatively enriched in the HB and SC). In all four tissues the top positively enriched pathways are associated with immune response. In the posterior tissues there is negative enrichment of some overlapping pathways with the anterior tissues, such as "DNA replication".

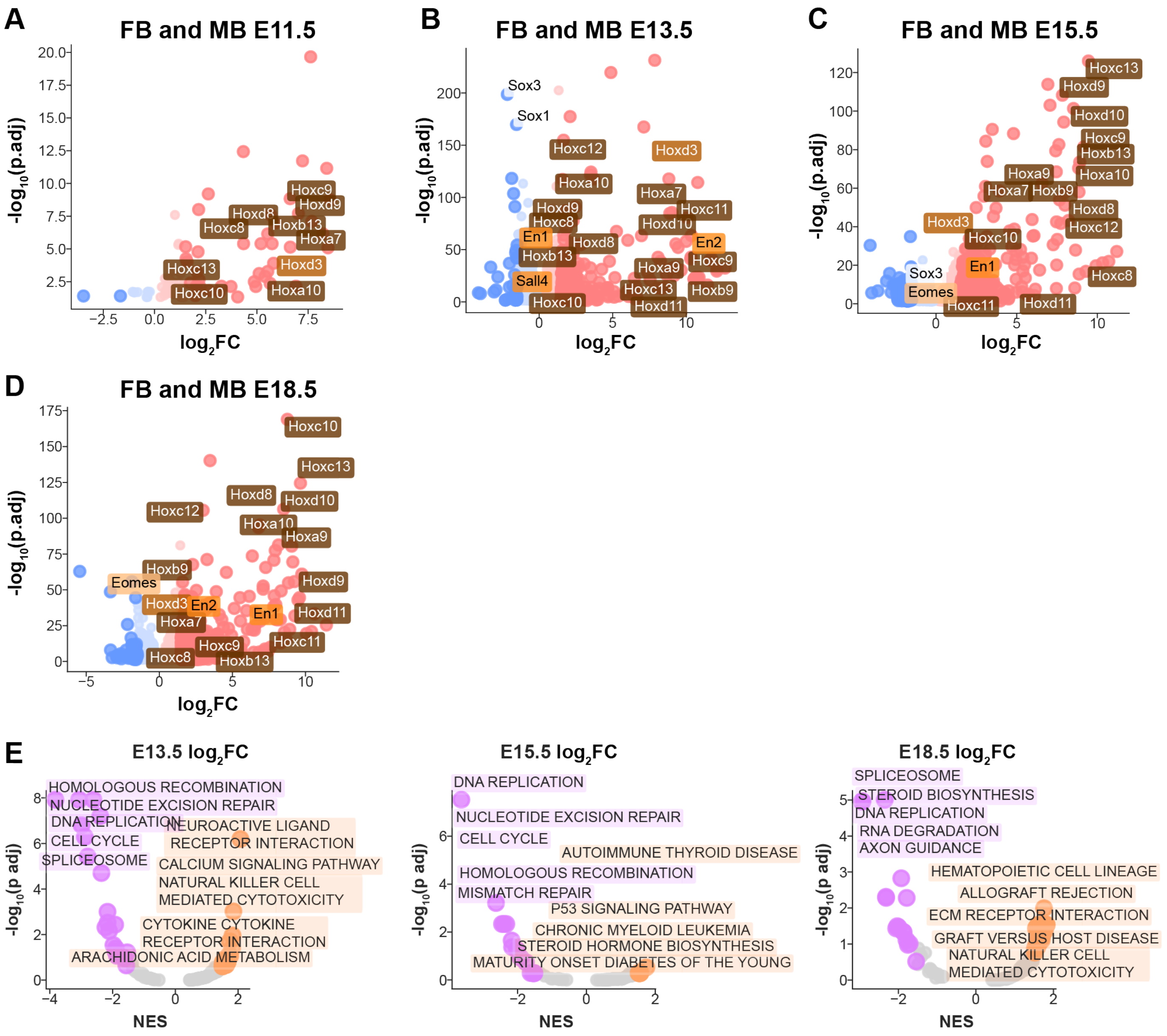

Supplemental Fig 2. Temporal response to *Eed-cKO* in anterior CNS (A) Anterior (FB, MB) regions at E11.5 shows limited upregulation of Hox genes and overall minor effects. (B) At E13.5, there is strong downregulation of progenitor markers *Sox3* and *Sox1* and upregulation of Hox genes. (C) At E15.5 there are fewer downregulated genes, however displaying a stronger upregulation response, in particular of Hox genes. (D) At E18.5 there is a similar upregulation as the earlier time points of Hox genes and strong downregulation of forebrain marker, *Eomes*. (E) At E13.5 and E15.5 there is downregulation of the "cell cycle" pathway. At E18.5 "cell cycle" is no longer a significant term but other terms such as "DNA replication" are shared. In the positively enriched pathways there is enrichment of immune associated pathways, and a response to a disrupted system (e.g., "allograft rejection")

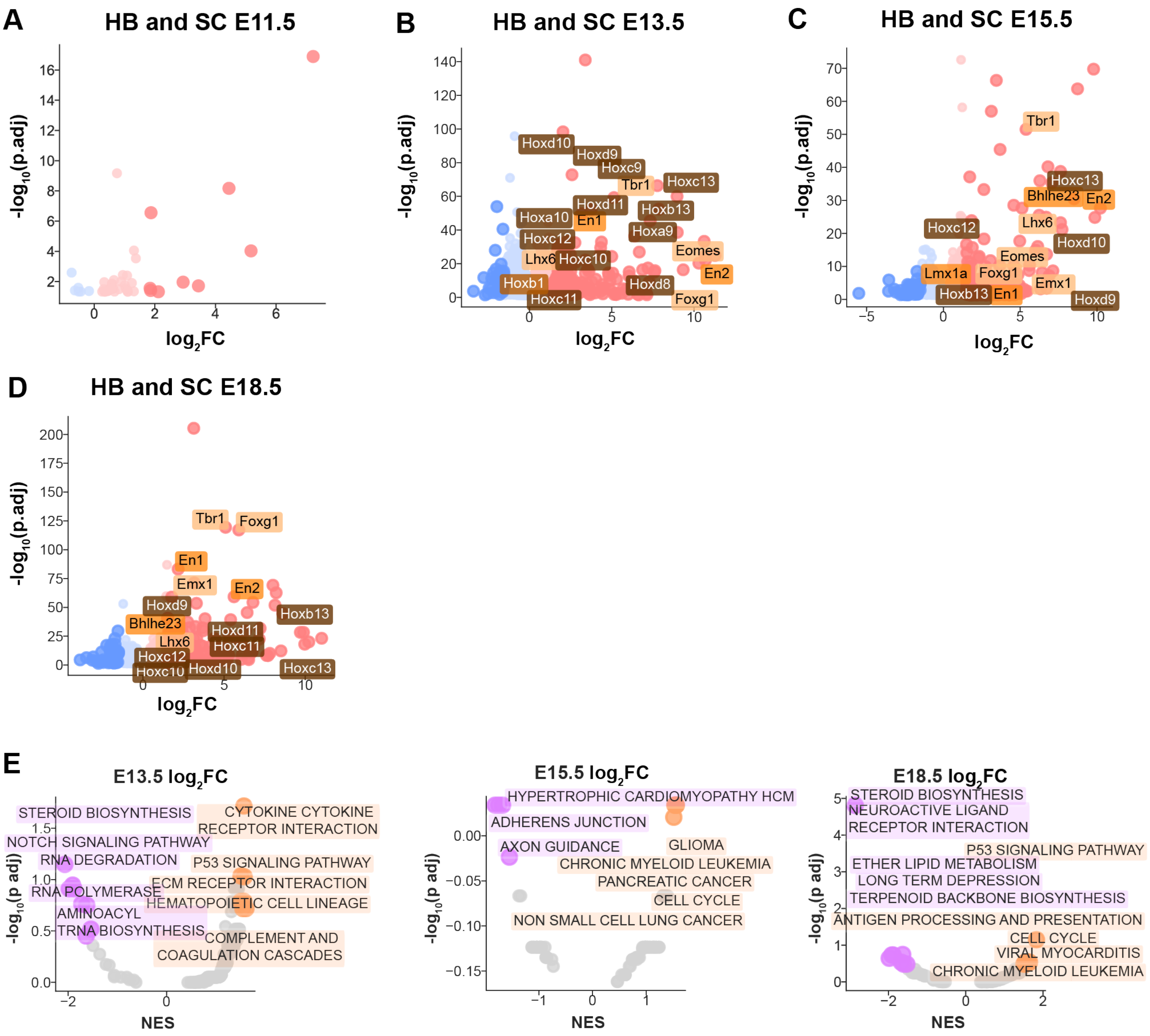

Supplemental Fig 3. Temporal response to *Eed-cKO* in posterior CNS (A) The $\log_2$FC in the posterior (HB, SC) regions at E11.5 shows minimal effects in *Eed-cKO*. (B) At E13.5 there is upregulation of both SC and FB markers. (C) At E15.5 there is upregulation of markers from across brain regions. (D) At E18.5 the same effects of ubiquitous upregulation are observed. (E) At E13.5 there is a downregulation of RNA pathways (“RNA degradation”, “RNA polymerase”), and an upregulation of immune response or cancer terms, as well as of “blood cells” and “hematopoietic cell lineage”. At E15.5 and E18.5 there is upregulation of the cell cycle pathway and also of blood cancer associated pathways, such as “chromic myeloid leukemia”. At the later stages there is negative enrichment for brain associated pathways, such as “neuroactive ligand receptor interaction” and “axon guidance”.

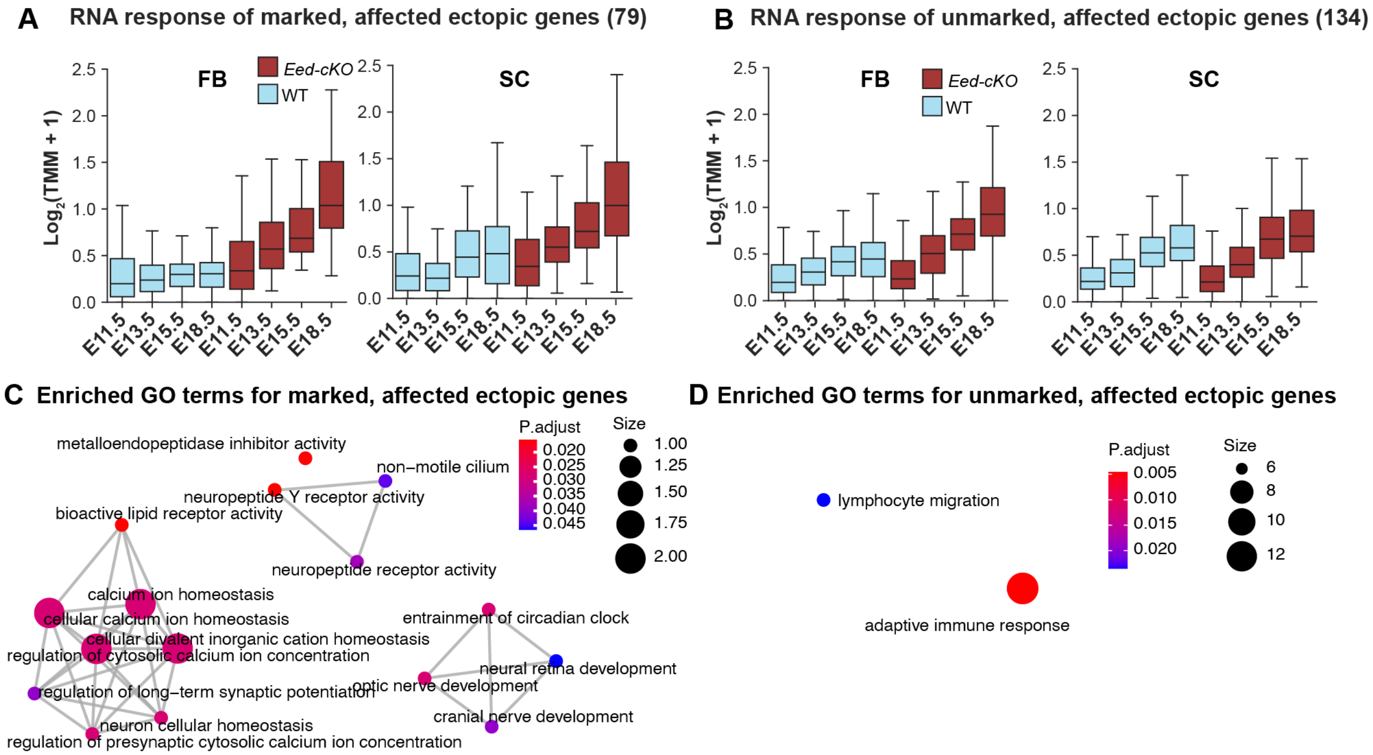

Supplemental Fig 4. (A) Marked peripherally expressed genes that were ectopically expressed in *Eed-cKO*, are upregulated over time in both FB and SC. (B) Unmarked peripherally expressed genes that were ectopically expressed in *Eed-cKO* are upregulated over time in FB, but with limited effects in SC. (C) GO analysis of marked genes shows a diverse range of terms. (D) Few terms were associated with the unmarked group.

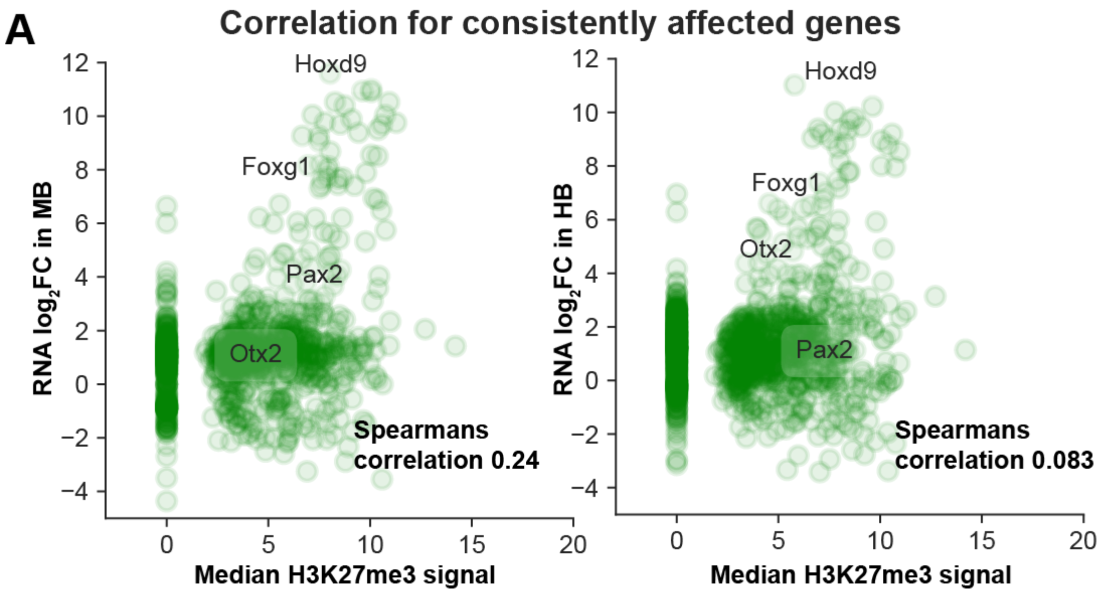

Supplemental Fig 5. MB $\log_2$FC and HB $\log_2$FC from the consistently affected gene category show minimal correlation with the median H3K27me3 signal across brain tissues.

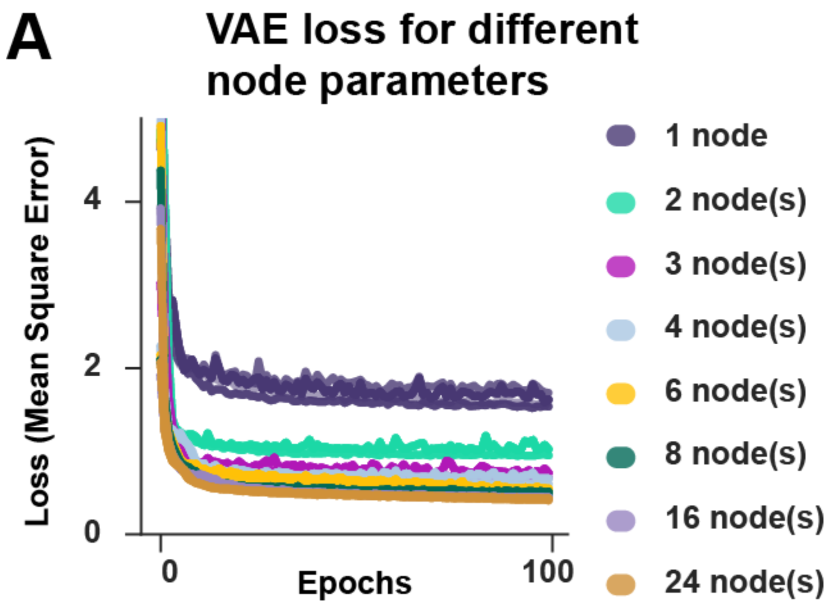

Supplemental Fig 6. Reconstruction loss of the VAE stabilises at 3 latent dimensions on the consistently affected dataset, with marginal improvements for greater numbers.

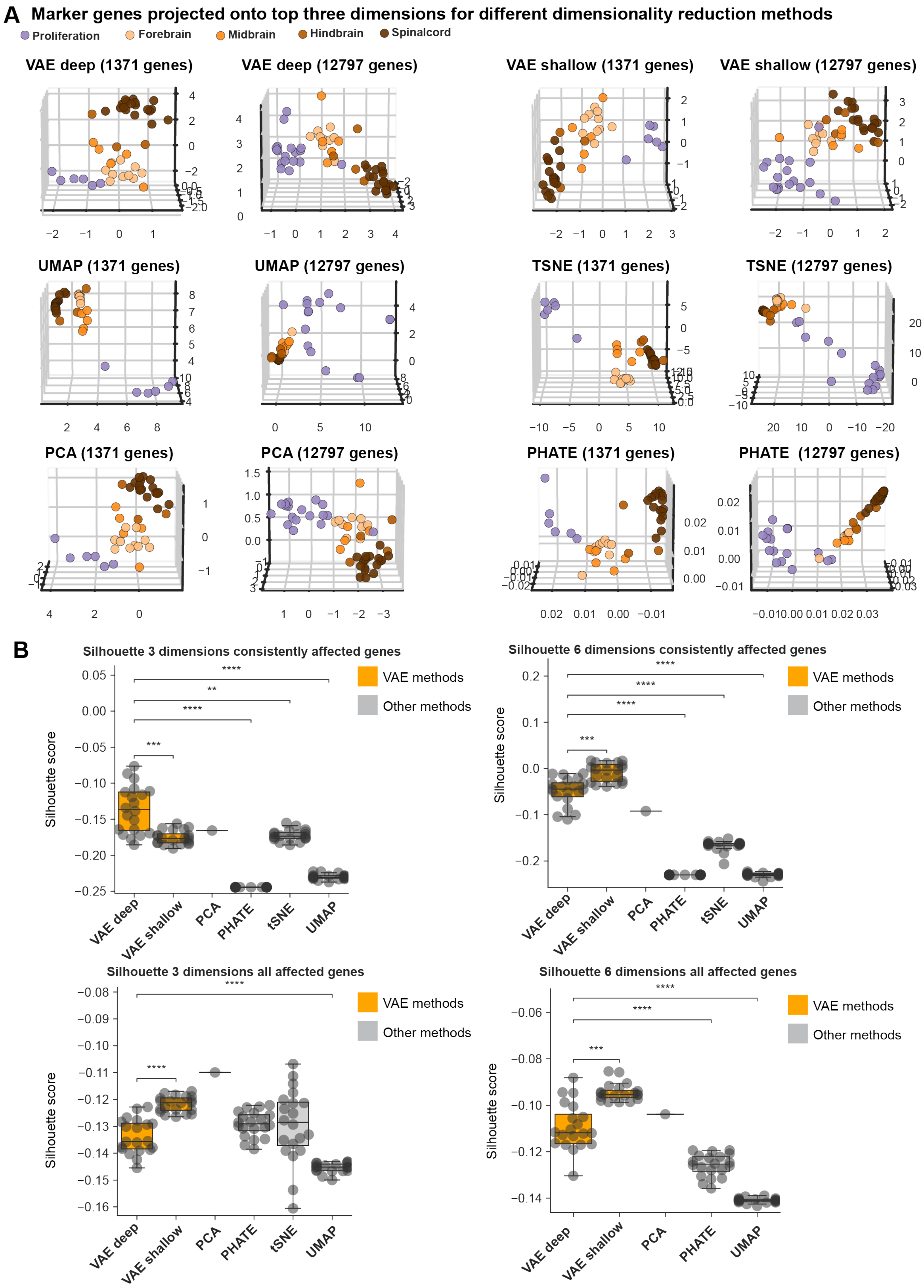

Supplemental Fig 7. Comparison between methods for reducing dimensionality of mixed gene profiles (A) Marker genes were projected onto 3-dimensional latent spaces produced by six methods: PCA, tSNE, UMAP, PHATE, a shallow VAE (a single internal layer) and a deep VAE (multi-layered as specified in Material & Methods). Groups of FB, MB, HB, and SC genes were most apparent when using the consistently affected gene set (1,371 genes) with separation reduced when adding the much larger set of partly affected genes. (B) Separability (as defined by Silhouette score, higher is better) between the diverse gene groups, each linked to a specific A-P development term, over 20 runs for tSNE, UMAP, deep VAE, shallow VAE, and PHATE and a single run for PCA (which is deterministic). tSNE was omitted from $D = 6$/all affected genes as it failed to complete (Ubuntu 20.04.2, 240GB System memory, Intel(R) Xeon(R) CPU E5-2690 v4 @ 2.60GHz) within 24 hours of runtime (all other tools completed within one hour).

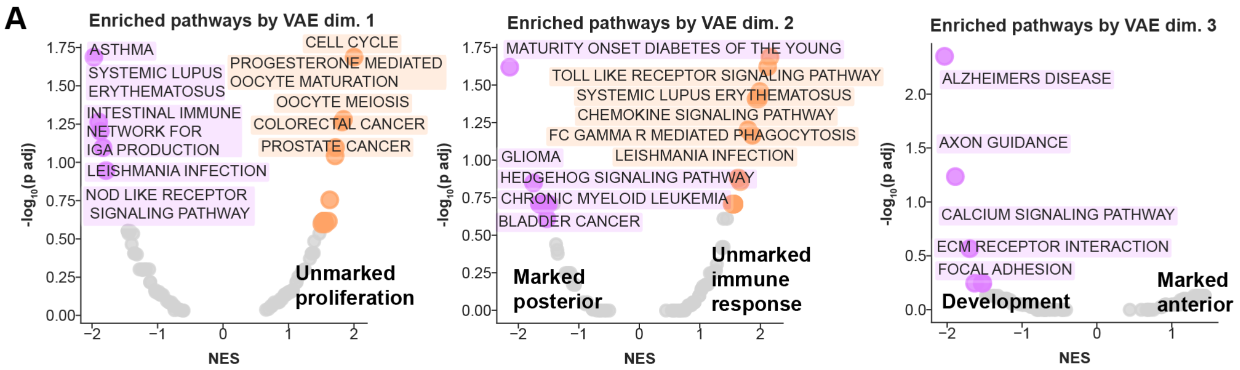

Supplemental Fig 8. GSEA for VAE Genes ordered by each VAE dimension enrich in KEGG pathways (top-5 shown as per NES), which concord with GO analysis (Figure 5) and reinforce the characterisation of each gene cohort, e.g., unmarked proliferation genes map to large tail of dimension 1, and intersect with pathways for “cell cycle” and “oocyte meiosis”.

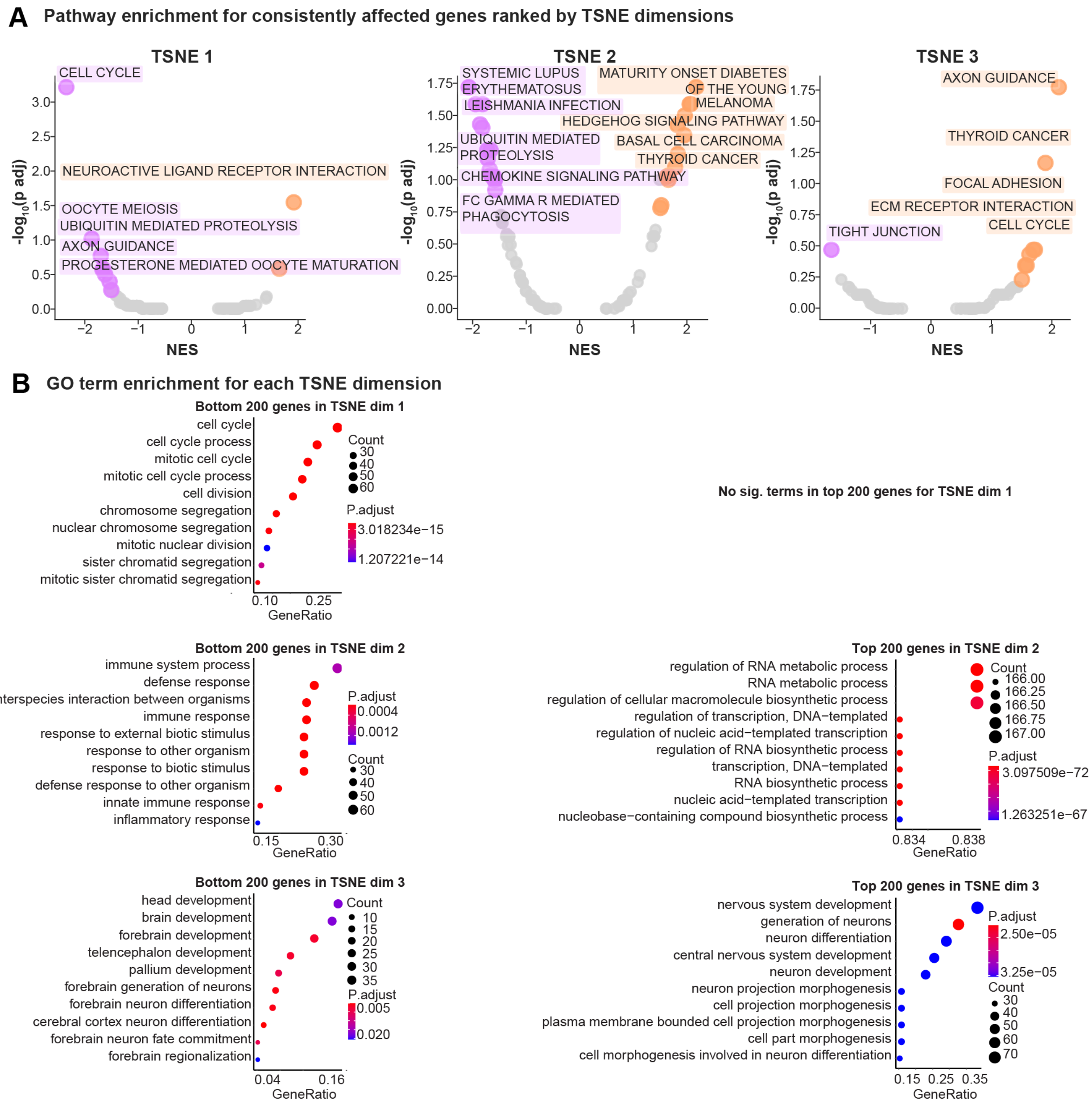

Supplemental Fig 9. Enrichment for tSNE (A) Genes were ranked by each tSNE dimension (up to 3) to identify negatively and positively enriched pathways. tSNE 1 is negatively enriched for cell cycle associated pathways and positively enriched for one pathway: "neuroactive ligand receptor interaction". tSNE 2 is negatively enriched for immune response pathways and positively enriched for cancer associated pathways. tSNE 3 is negatively enriched for one pathway; "tight junction", while positively enriched for diverse terms, including "cell cycle" and brain pathways. (B) The top and bottom 200 genes along each tSNE dimension were tested for enriched GO terms. tSNE 1 aligns with cell cycle associated terms, fitting with the enriched pathways along dimension 1, while not enriched for any terms in the top genes. In dimension 2, the bottom genes enrich immune response genes, and the top RNA metabolism associated GO terms (contain the Hox genes), all of which agree with enriched pathways. The terms for bottom genes in tSNE 3 overlap with forebrain development, while the terms for the top are more diverse, analogous to pathways, enriching for cell projection as well as development terms. None of the gene cohorts appear to specifically encode for development terms.

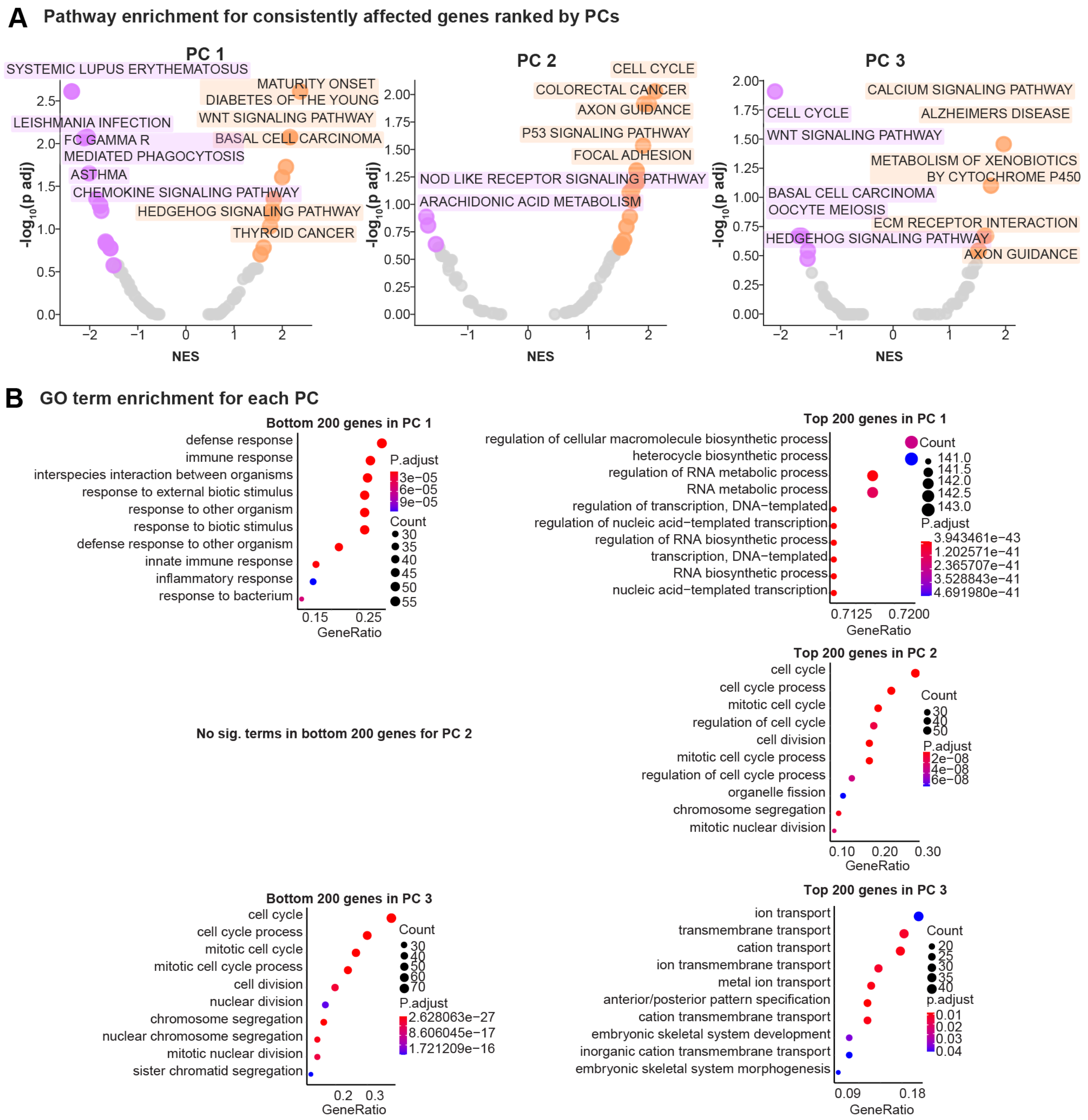

Supplemental Fig 10. Enrichment for PCA (A) Genes were ranked by each Principal Component (PC) (up to 3) to identify negatively and positively enriched pathways. PC 1 is negatively enriched for immune associated pathways, and positively enriched for brain dysregulation, "WNT signalling, Hedgehog signalling" and cancer pathways. PC 2 is negatively enriched for only two pathways: "Nod like receptor" and "arachidonic acid metabolism", which is associated with neurotransmitter systems. The top five positive pathways in PC 2 are diverse, including "cell cycle", "axon guidance" and cancer pathways. PC 3 is negatively enriched for diverse pathways, including cell cycle, carcinoma, and Hedgehog signalling. PC 3 is positively enriched for brain associated pathways, including "Alzheimers" and "axon guidance". (B) The top and bottom 200 genes along each PC were tested for enriched GO terms. PC 1 agrees with the enrichment of pathways, e.g., with RNA metabolism associated GO terms positively enriched (contain Hox genes). In PC 2 the top 200 genes are not enriched for any GO terms, while the bottom 200 enriched predominately for cell cycle terms. PC 3 is negatively enriched for similar terms that PC 2 enriched for, and positively enriched for transport associated and development terms. There is no enrichment for anterior specific function.

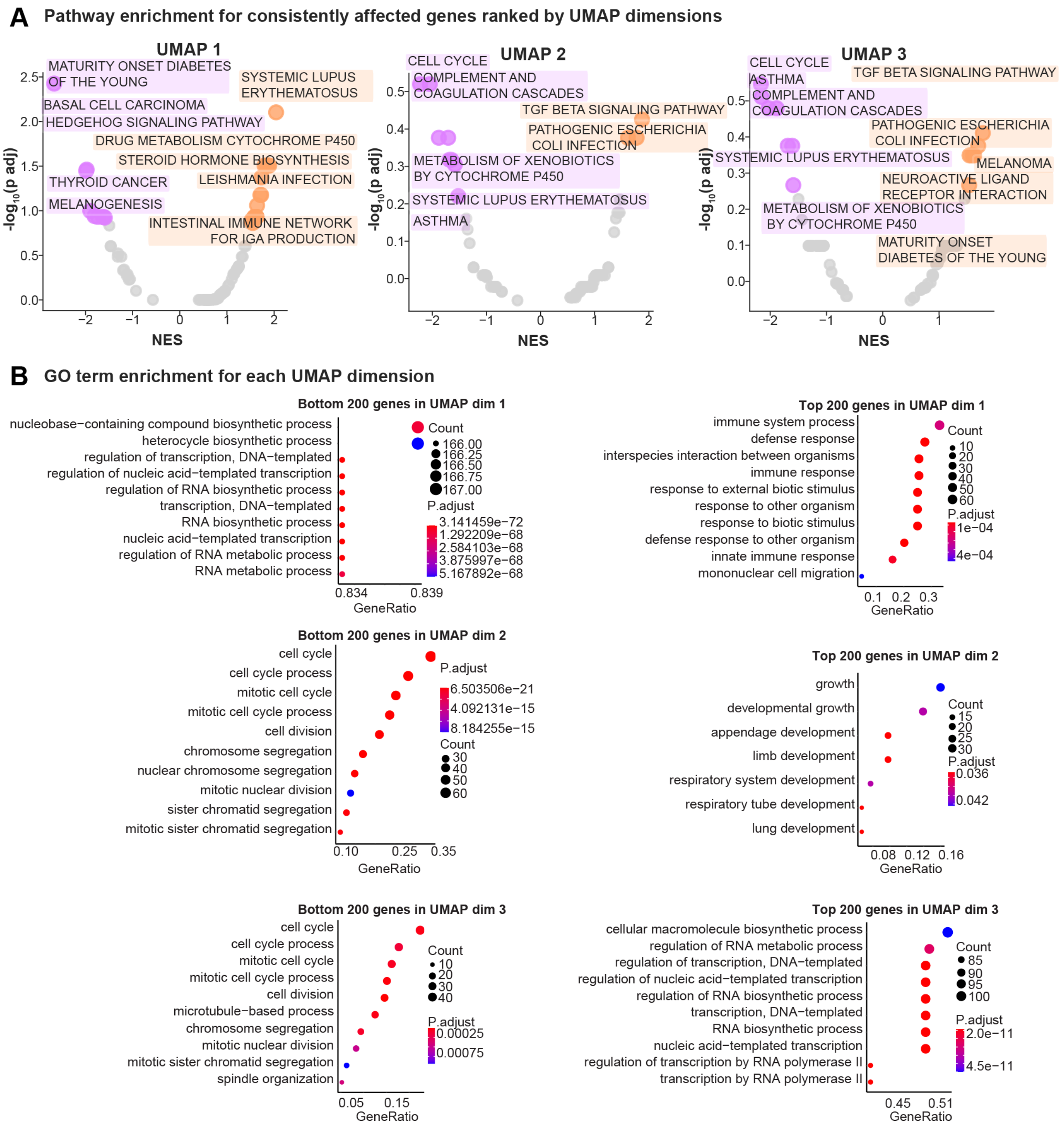

Supplemental Fig 11. Enrichment for UMAP (A) Genes were ranked by each UMAP dimension (up to 3) and to identify negatively and positively enriched pathways. UMAP 1 is negatively enriched for cancer associated pathways, and positively enriched for immune response. UMAP 2 is negatively enriched for a diverse range of pathways, including "cell cycle" and the immune associated "systemic lupus erythematosus". UMAP 2 is only enriched for two pathways: "TGF beta signalling", which is associated with development, and "pathogenic Escherichia coli infection". The top five positive pathways in UMAP 3 covers similar pathways to UMAP 2 i.e., "cell cycle" and immune associated pathways. UMAP 3 is positively enriched for terms from UMAP 1 and UMAP 2. (B) The top and bottom 200 genes along each UMAP dimension were tested for enriched GO terms. UMAP 1 is positively enriched with RNA metabolism associated GO terms (contain the Hox genes), which does not overlap strongly with the enriched pathways. The top genes from UMAP 1 predominantly enrich in immune response terms and agree with pathways. The bottom genes from UMAP 2 are associated with cell cycle terms, while the opposing side of the dimension are development associated. In UMAP 3 both bottom and top terms overlap with UMAP 2. There is no enrichment for anterior specific function.

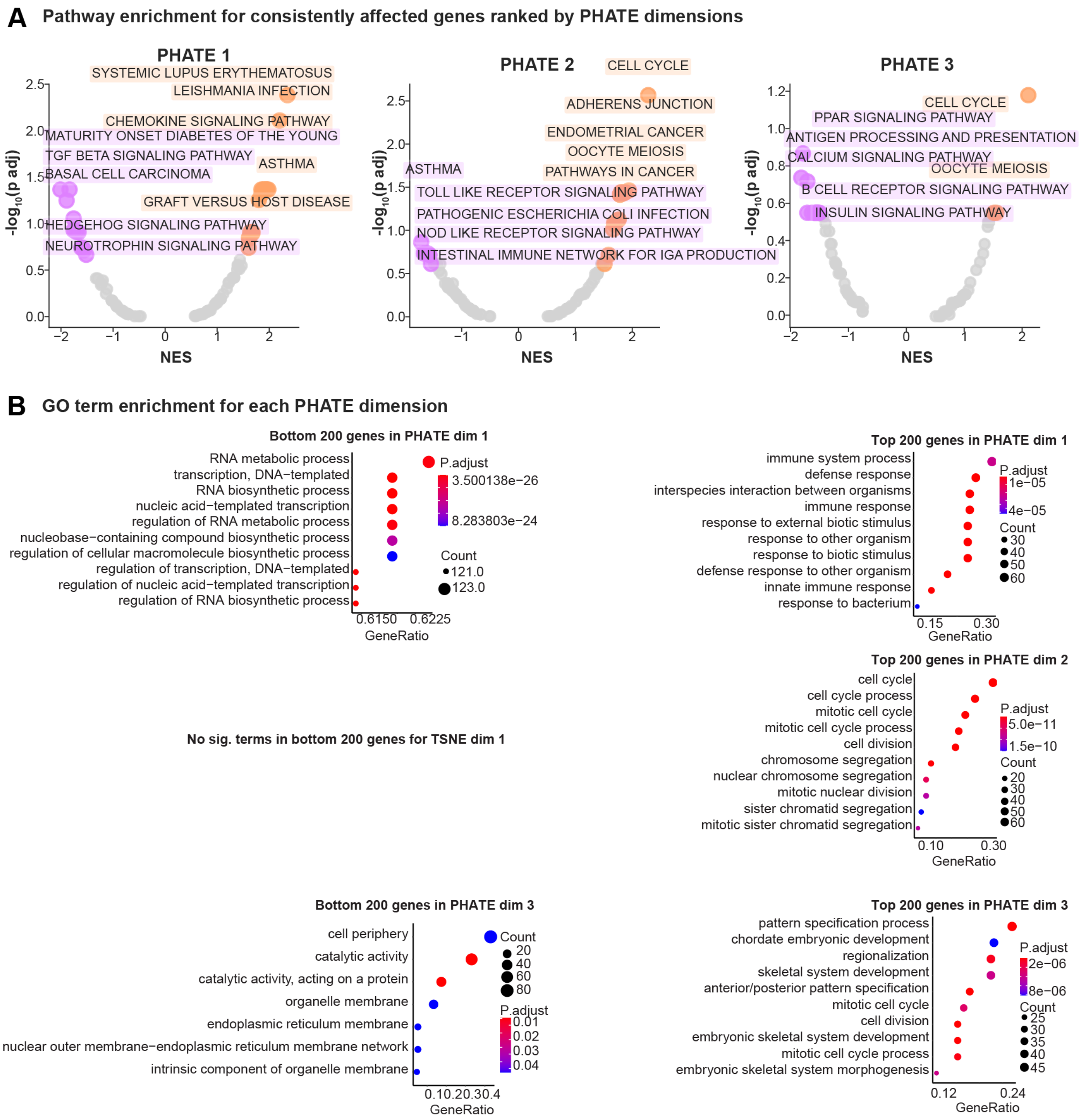

Supplemental Fig 12. Enrichment for PHATE (A) Genes were ranked by each PHATE dimension (up to 3) to identify negatively and positively enriched pathways. PHATE 1 is negatively enriched in cancer and brain associated pathways and positively enriched in immune associated pathways. PHATE 2 is negatively enriched in immune response pathways and positively enriched in cancer and cell cycle associated pathways. PHATE 3 is negatively enriched in signalling pathways and positively enriched in cell cycle pathways. (B) The top and bottom 200 genes along each PHATE dimension were tested for enriched GO terms. PHATE 1 bottom genes enrich for RNA metabolism associated GO terms (contain the Hox genes), the top genes of this dimension are enriched for immune response. In dimension 2 the top genes enrich for cell cycle terms similarly to the pathways in (A). The bottom genes from PHATE 3 overlap with cell periphery, and membrane terms, these terms are not enriched in any of the other methods as such would be a unique group. The top genes are associated with development, which does not overlap with the most significant pathways (cell cycle). There is no enrichment of the groups for anterior specific function.

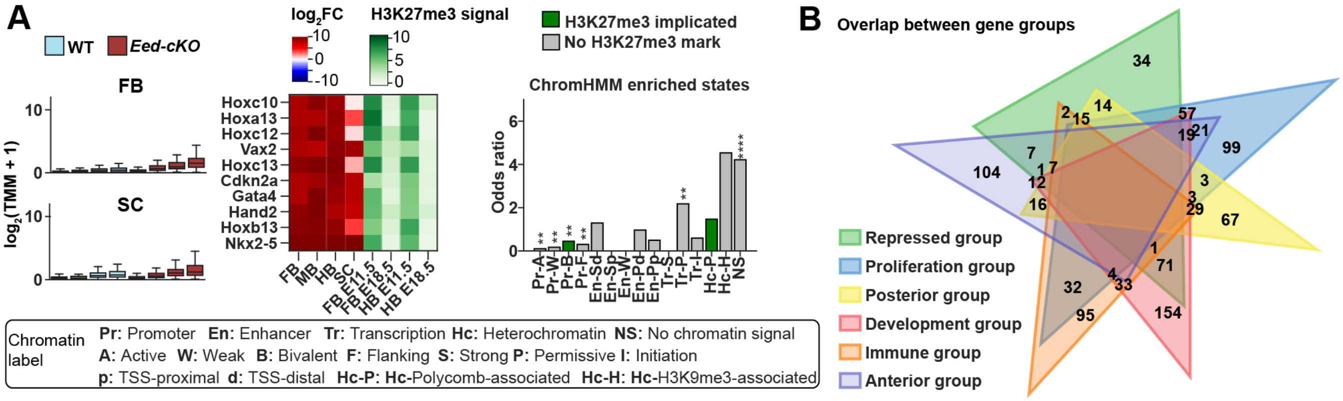

Supplemental Fig 13. Results for repressed cohort (A) Genes from the repressed gene cohort show overall low gene expression in both FB and SC. They are marked and enriched for the no-signal (NS) ChromHMM chromatin state. (B) Overlap between extreme groups of genes identified by the VAE shows that the repressed group strongly overlaps with the posterior group.

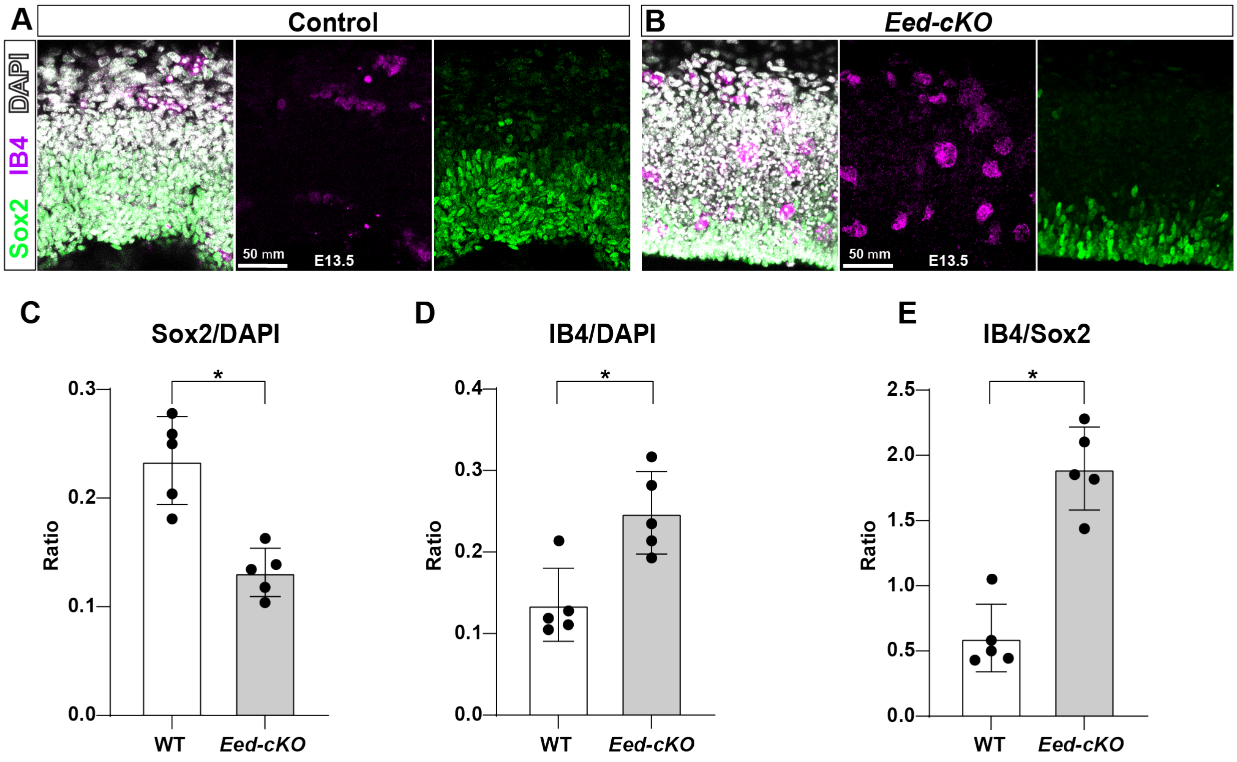

Supplemental Fig 14. IB4 staining reveals increased ratio of immune cells and blood vessels to CNS tissue in *Eed-cKO*. (A-B) Immunostaining for Sox2 (progenitors), IB4 (microglia and blood vessels) and DAPI (nuclei) in the WT and *Eed-cKO* telencephalon at E13.5. Deletion of *Eed* results in a reduced progenitor domain and an increase in IB4 staining in the CNS. (C-E) Quantification of Sox2/DAPI, IB4/DAPI and IB4/Sox2 ratios (mean with SD; $n \geq 5$ scans per genotype and stage; $p \leq 0.05$ (*)).

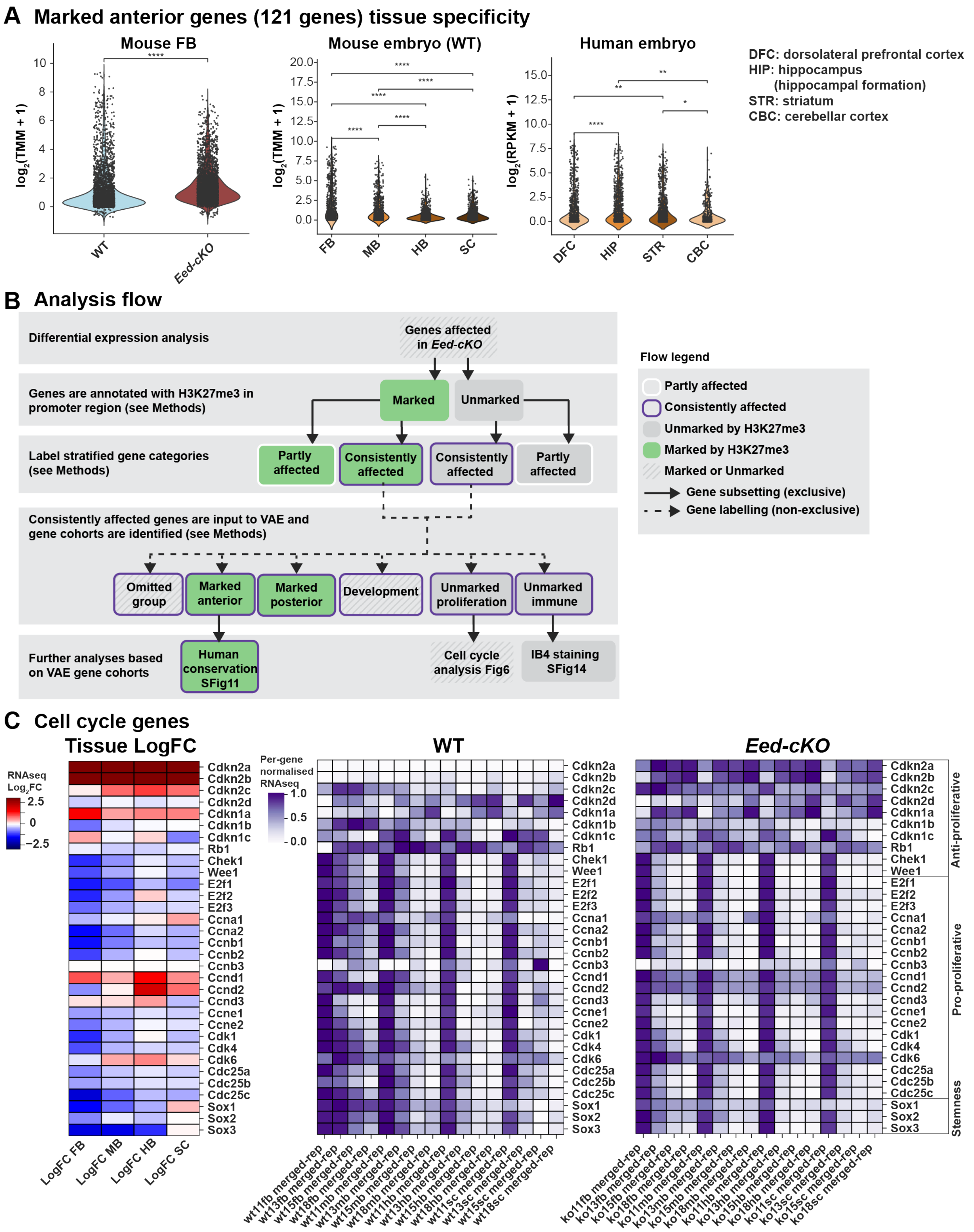

Supplemental Fig 15. Analyses based on VAE gene cohorts (A) The 121 genes mapping to the anterior marked group show conserved tissue specific trends across human (77 human homologs, along time points W1-W4) and mouse data (E13.5-E18.5). (B) Flow diagram of genes used in the analyses, from differentially expressed genes, to considering H3K27me3, and sorting by VAE into cohorts. (C) Pro-proliferative, anti-proliferative, and stemness gene profiles for log fold change across tissues and the profile for WT and *Eed-cKO*.
